# **Genetic variation and metabolic pathway intricacy govern the active compound content and quality of the Chinese medicinal plant*Lonicera japonica*thunb**

**DOI:** 10.1186/1471-2164-13-195

**Published:** 2012-05-20

**Authors:** Yuan Yuan, Lipu Song, Minhui Li, Guiming Liu, Yanan Chu, Luyu Ma, Yuanyuan Zhou, Xiao Wang, Wei Gao, Shuangshuang Qin, Jun Yu, Xumin Wang, Luqi Huang

**Affiliations:** 1 Beijing Key Laboratory of Functional Genomics for Dao-di Herbs,Institute of Chinese Materia Medica, Academy of Chinese Medical Sciences, Beijing, 100700, China; 2 CAS Key Laboratory of Genome Sciences and Information, Beijing Institute of Genomics, Chinese Academy of Sciences, Beijing, 100029, China; 3 Shandong Academy of Medical Sciences, Jinan, 250031, China; 4 Baotou Medical College, Baotou, 014040, China; 5 Shandong Analysis and Test center, Jinan, 250014, China

**Keywords:** RNA-seq, Transcriptome, Active compounds, Synthetic pathways, *Flos Lonicerae Japonicae*

## Abstract

**Background:**

Traditional Chinese medicine uses various herbs for the treatment of various diseases for thousands of years and it is now time to assess the characteristics and effectiveness of these medicinal plants based on modern genetic and molecular tools. The herb *Flos Lonicerae Japonicae* (FLJ or *Lonicera japonica* Thunb.) is used as an anti-inflammatory agent but the chemical quality of FLJ and its medicinal efficacy has not been consistent. Here, we analyzed the transcriptomes and metabolic pathways to evaluate the active medicinal compounds in FLJ and hope that this approach can be used for a variety of medicinal herbs in the future.

**Results:**

We assess transcriptomic differences between FLJ and *L. japonica* Thunb. var. chinensis (Watts) (rFLJ), which may explain the variable medicinal effects. We acquired transcriptomic data (over 100 million reads) from the two herbs, using RNA-seq method and the Illumina GAII platform. The transcriptomic profiles contain over 6,000 expressed sequence tags (ESTs) for each of the three flower development stages from FLJ, as well as comparable amount of ESTs from the rFLJ flower bud. To elucidate enzymatic divergence on biosynthetic pathways between the two varieties, we correlated genes and their expression profiles to known metabolic activities involving the relevant active compounds, including phenolic acids, flavonoids, terpenoids, and fatty acids. We also analyzed the diversification of genes that process the active compounds to distinguish orthologs and paralogs together with the pathways concerning biosynthesis of phenolic acid and its connections with other related pathways.

**Conclusions:**

Our study provides both an initial description of gene expression profiles in flowers of FLJ and its counterfeit rFLJ and the enzyme pool that can be used to evaluate FLJ quality. Detailed molecular-level analyses allow us to decipher the relationship between metabolic pathways involved in processing active medicinal compounds and gene expressions of their processing enzymes. Our evolutionary analysis revealed specific functional divergence of orthologs and paralogs, which lead to variation in gene functions that govern the profile of active compounds.

## Background

*Flos Lonicerae Japonicae* (FLJ, *Lonicera japonica* Thunb.) is used as a herbal medicine with anti-inflammatory effect [1]. The first record in the literature on this herb is found in one of the world earliest pharmacopoeias, the Shen-Nong’s Herbals. The commercial value of FLJ in herbal medicine trading markets has increased over 400% in recent years, and over 30% of current traditional Chinese medicine prescriptions contain FLJ. This important herb is used to treat various diseases, such as severe acute respiratory syndromes, H1N1 influenza, and hand-foot-and-mouth disease. FLJ extracts also show other biological and pharmaceutical properties, including, anti-bacterial, anti-inflammatory, anti-viral, liver protection [2], anti-angiogenic, and antinociceptive activities [3]. However, the quality of FLJ as a medicinal herb is rather inconsistent and largely due to its uncharacterized active compound content.

Chlorogenic acid and luteoloside are biomarkers used by the Chinese Pharmacopoeia (Chinese Pharmacopoeia Commission, 2010) for evaluating the quality of FLJ. Pharmacological experiments show that luteolin has a spectrum of biological activities, particularly antioxidative and anti-inflammatory properties. Luteolin has a direct inhibitory effect on lung fibrosis [4]. Other phenolic compounds, including phenolic acids, have been identified in the methanolic extract of FLJ by liquid chromatography with time-of-flight mass spectrometry [5]. Among these compounds, loganin and sweroside also exhibit anti-inflammatory and analgesic activities, which are candidate active components of the FLJ extract [6]. Rutin is demonstrated to reduce oxidative stress-mediated myocardial damage in both *in vitro* and *in vivo* models and may prove beneficial in the treatment of myocardial infarction [7].

FLJ has other commercial applications, such as scent additive used in foods and cosmetics [8]. The main volatile component is linalool, but other floral volatile compounds, including germacrene D, *cis*-jasmone, E,E-α-farnesene, nerolidol, *cis*-3-hexenyl acetate, hexyl acetate, *cis*-hexenyl tiglate, and indole, have been detected based on headspace analysis and gas chromatography–mass spectrometry (GC-MS) [9].

The concentrations of active components and volatile compounds are closely correlated with floral developmental stages [10]. The content is higher in the early stages of cell differentiation status and the flower buds often show the highest medical value, whereas the chlorogenic acid content is significantly reduced in blooming flowers. However, the budding period is both short and not synchronized among individual plants, making it problematic for mass harvest.

A limited number of publications have assessed the relationship between the abundance of active compounds and floral development of FLJ based on molecular biological techniques. One of the studied showed that hydroxycinnamoyl-CoA quinate hydroxycinnamoyl transferase (HQT) gene, encoding a protein of 439 amino acids and identified in FLJ, has a tissue distribution that correlated with the pattern of chlorogenic acid abundance [11]. Another study cloned multi-copied allene oxide synthase LjAOS (GenBank accession: DQ303120) from FLJ and demonstrated that LjAOS mRNA is most abundant in flower buds, and its expression correlates with the concentration of chlorogenic acid [12].

The content of active compounds also differs significantly among the species and varieties [13] of the Lonicerae family. *L. japonica* Thunb. var. chinensis (Watts) (rFLJ) is a Chinese local variety and its corolla has purple outer (upper) and white inner (lower) portions and the whole flower has different active compound contents when compared with FLJ [14]. Qin *et al.*[15] reported different chlorogenic acid contents between FLJ and rFLJ. Changes of the active compound contents often result in different pharmacological activity and medicinal quality [16]. However, a systematic study to compare gene expression and active compound variations in the active parts among varieties and closely related plant species is of essence.

The high-throughput of the next generation RNA sequencing (RNA-seq) technologies offers rapid genome-wide transcriptomic studies and is widely used to define gene structure and expression profiles in model organisms [17-19]. The assembly of *de novo* transcriptomes based on short reads generated from RNA-seq method allows gene discovery in organisms without reference genomes. In this study, we applied RNA-seq to the study of floral transcriptomes of FLJ and rFLJ. We generated over 100 million reads using the Illumina GAII platform, and analyzed over 6,000 expressed genes from each of the three flowering stages: bud, blooming, and bloomed. We correlated the transcriptomic profiles with metabolic activities of the relevant active compounds, including phenolic acids, flavonoids, terpenoids, and fatty acids, to elucidate effects of enzymatic divergence on biosynthetic pathways.

## Methods

### Plant materials

**Figure 1:**
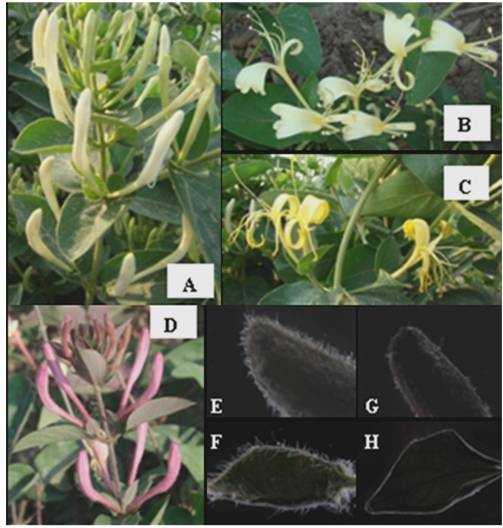
**Plant materials.** (A) –(D), Flower samples: A, FLJ bud, had white petals and had not yet bloomed into a full-size flower; B, FLJ flower1, had white petals and had bloomed into a full-size flower; C, FLJ flower2, had yellow petals and had bloomed into a full-size flower; D, rFLJ bud, had red petals and had not yet bloomed into a full-size flower. (E) –(H), Stereomicroscopic detection Trichome (red arrows) in the flower bud and leaf of FLJ and rFLJ. Abbreviations: E, FLJ flower bud; F, FLJ Leaf; G, rFLJ flower bud; and H, rFLJ leaf.

Flower samples (corollas or all petals) were randomly collected five each from 3-year old FLJ and rFLJ in Doudian plantation (Beijing, China) (Figure 1). The flowering stages are: (1) the bud stage (white, FLJ and red, rFLJ) when the flower bud has not bloomed into a full-size flower yet; (2) the flower1 stage when the white inner petals and white (FLJ) or red (rFLJ) outer petals has just bloomed into full-size flowers; and (3) the flower2 stage when the yellow inner petals and white (FLJ) or red (rFLJ) outer petals bloomed into full-size flowers. We separated the samples into 2 groups; group 1 is used to compare the FLJ flower buds with its flowers from flower1 and flower2 stages, and group 2 is used to compare the flower buds between FLJ and rFLJ. Fresh samples were used for gas chromatography–mass spectrometry (GC-MS), and freeze-dried flowers were used for HPLC. Quick-frozen flowers (in liquid nitrogen) were used for RNA extraction.

### RNA isolation and sequence acquisition

Total RNA was extracted from flower samples by using Concert Plant RNA Reagent (Invitrogen, Cat. 12322–012) according to the manufacturer’s protocol. RNA integrity was measured by using gel electrophoresis and spectrophotometer (Nonodrop). An Oligotex-dT30 Super mRNA Purification Kit from TaKaRa (D9086) was used to extract mRNA.

### De novo sequence assembly and contig clustering

Prior to assembly and mapping, we removed low quality reads (such as >30% “N” in a read and polyA tails) from the raw data and assembled the processed reads into contigs using ABySS http://www.bcgsc.ca/platform/bioinfo/software/abyss; [20]. We used contigs longer than 100 bp for further annotations. Since the genome sequence of FLJ has not been available, we used BLAST [21] to align the contigs to the NCBI non-redundant sequence database. Because *V. vinifera* full-length cDNA sequences provided the most annotations, we clustered the FLJ/rFLJ contigs in reference to the *Vitis vinifera* cDNA sequences.

### Gene annotation and expression analysis

We used BLASTX [21] to search against the NCBI non-redundant (nr) database to identify transcripts and annotated the transcripts using KEGG [22] and COG [23] with an E value cut-off of 10^−5^. We applied InterPro [24] and Blast2GO [25] to the annotation of protein motifs/domains and Gene Ontology (GO) terms. GO annotation enrichment analyses were conducted based on a Benjamini and Hochberg false discovery rate correction with significance set at *p* < 0.05 by using the Cytoscape plug-in BiNGO[26].

We mapped the sequence reads and contigs using SOAP http://soap.genomics.org.cn/soapaligner.html; [27] and handled isoforms/spliced variants with cautions [28]. We used sequence similarity information and the *Vitis vinifera* full-length cDNAs for transcriptome mapping and tag counting using LASTZ [29] after clustering the contigs into ESTs. Only uniquely mapped reads were counted. The expression profiling was done by normalizing the total mapped reads and contig length as RPKM (reads per kilobase of transcript sequence per million mapped reads; 19). The effective size was used to adjust RPKM values in subsequent analyses. DEGseq1.2.2 [30] was used to roughly identify the differentially expressed genes (DEGs) *via* the *p* value and the RPKM fold-change value. The DEGs were further studied based on pathway/expression analyses and real-time PCR.

### Gas chromatography–mass spectrometry profiling

The concentrations of ethanol, acetate, alkane, and terpene in the flower samples were determined based on gas chromatography–mass spectrometry (GC-MS) ( Additional file 1: Figure S1). Fresh flower samples (1.00 g, n = 3) were washed twice with distilled water, subjected to ultrasonic extraction (90 Hz) with 10 ml ethyl acetate for 40 minutes, and filtered through a microfiltration membrane (0.45 μm). Extracted metabolites were analyzed as follows: 1 μl of sample was injected at a split ratio of 10:1 into a Shimadzu GCMS-QP2010 instrument. A VF-5MS capillary column coated with 5% phenyl and 95% dimethylpolysiloxane (30 m × 0.25 mm i.d. and 0.1 μm film thickness; Varian, USA) was employed for separation. Injection temperature was 230°C and the interface temperature was set to 250°C. The ion source was adjusted to 230°C and the solvent cut-time was set to 3 minutes. Helium was the carrier gas at a flow-rate of 1.05 ml minute^−1^. The temperature program was: an initial temperature of 50°C, programmed at 5°C minutes^−1^ to 150°C and held for 10 minutes, then ramped at 10°C minute^−1^ to 260°C and held for 20 minutes. The mass spectrometric detector was operated in the electron impact ionization mode with an ionizing energy of 70 eV, scanning from 40–400 m/z. Peak identification was performed by employing AMDIS (NIST, Gaithersburg, MD, USA) and WILEY7n (Palisade Corporation, NY, USA) databases with a spectral match quality >90%. An internal standard of pentadecanol was added to correct for differences in derivatization efficiency and changes in sample volume during heating. Peaks were quantified by area integration and concentrations were normalized to the quantity of the internal standard recovered. Two technical replicates were analyzed for three biological samples from each flowering stage.

### HPLC Profiling

The dried flowers were separately comminuted with a miller. Each solid sample (40 mesh, 0.50 g) was accurately weighed and extracted with 50 ml of 70% aqueous ethanol by ultrasonication for 30 minutes. The extract was cooled to 25°C and diluted to 50 ml with 70% aqueous ethanol, filtered with a 0.45 μm Millipore filter membrane. Then, 10 μl of the filtrate was injected into the HPLC system for analysis ( Additional file 1: Figure S2).

The HPLC system was an Agilent 1200LC series (Agilent Technologies, Palo Alto CA, USA), consisting of an online vacuum degasser (G1379B), a Bin pump SL (G1312B), an auto-sampler (GB67C), a thermostatic column compartment (G1316B), and a diode-array detector (DAD) (G1315C) coupled with an analytical workstation. The column configuration was an Agilent TC-C18 reserved-phase column (5 μm, 250 mm × 4.6 mm). The sample injection volume was 10 μl. The detection wavelength was set at 242 nm for analysis with the flow rate at 1.0 ml minute^−1^, and the column temperature remaining at 25°C. The mobile phase contained deionized water, formic acid (A; 99:1, v/v), and methanol (B). The elution conditions are shown in Additional file 2: Table S1. To determine the linearity of the chromatographic techniques, calibration plots of eight standards were constructed on the basis of peak areas (*y*) using seven different concentration solutions ( *x*). All plots were linear in the examined ranges, and linear ranges were shown at different concentrations for the standard compounds (μg ml^−1^). The *r* value in Additional file 2: Table S2 refers to the correlation coefficient of the equation for calculating the content of compound. All the standard compounds showed good linearity (r > 0.9994) in a relatively wide concentration range. The standard compounds, chlorogenic acid (110753), caffeic acid (110885), ferulic acid (110773), rutin (100080), huteoloside (111720), Hyperoside (111521), quercitrin (111538), and quercetin (100081), were purchased from National Institutes for Food and Drug Control in China.

### Principal component analysis and statistical assessment of GC-MS and HPLC data

Data sets containing more than two independent biological replicates per samples were statistically analyzed based on the Student’s *t* test with a significance cutoff of *p* < 0.05. To assess the metabolic changes or differences between samples and to identify metabolic changes involved in group discrimination, multivariate analyses (PCA, PLS-DA) were performed by using the SIMCA-P + (12.0.0.0.0) program (Umetrics AB, Tvistevdgen 48 Umea 907 19, Sweden).

### Phylogeny and identification of paralogs and orthologs

We used the PFAM database [31] for validating all the gene families and protein sequences and constructed neighbor-joining trees for all sequences (ClustalW2). To identify orthologs, we performed an all-against-all sequence comparison using BLAST (cutoff <10^-20^) and determined orthologs from the best reciprocal hits >80% alignment length; [32].

### Experimental validation of transcribed sequences

We used RNA samples extracted from the flower samples of FLJ and rFLJ to perform qRT−PCR and M-MLV reverse transcriptase cDNA Synthesis Kit from Takara. The PCR primers are shown in Additional file 2: Table S5. The amplification condition was set as follows: initiated by 1-minute incubation at 95°C, followed by 35 cycles at 95°C for 15 seconds, 57–60°C for 30 seconds, and 68°C for 30 seconds. PCR results were evaluated by using 2–3% NuSieve agarose gels.

## Results

### Paired-end sequencing and *de novo* assembly

We designed a paired-end sequencing strategy and acquired nearly-saturated raw sequence data for all five libraries, including FLJ bud, FLJ flower1, FLJ flower2, rFLJ bud, and rFLJ flower2 in a range of 27–41 million reads per library (Table 1). After quality filtering and assembly, the usable sequence reads per library totaled 13–32 million reads. Given the read lengths of either 76 bp or 81 bp, the net transcriptome coverage is deemed adequate.

**Table 1:** Summary of sequencing and assembling data

**Samples**	**Reads**		**Average Read length(bp)**	**Number of contigs**	**Average contigs length(bp)**	**N50 Of contigs**	**Mapped reads**	**Expressed Genes**
	**Raw**	**Processed**						
FLJ B	32265956	24849381	76	32171	763	1394	11434981	6218
FLJ F1	30007720	15279615	81	41794	412	681	8602791	6088
FLJ F2	41392696	31858918	76	41608	544	1077	17927893	6591
rFLJ B	31021220	16497776	81	25232	692	1214	8943545	5837
rFLJ F2	26778792	12943328	81	39415	552	1093	4697897	5330

***Abbreviations:*** FLJ, *Lonicera japonica* Thunb; rFLJ, *Lonicera japonica* Thunb. var. chinensis (Watts.); B, bud; F1, flower1; and F2, flower2.

We employed ABySS (http://www.bcgsc.ca/platform/bioinfo/software/abyss; 20), an assembler developed specifically for the next-generation short-read sequences, to assemble the processed sequences and obtained a total of 180,220 contigs, ranging from 25,232 to 41,796 for each library. We assembled all reads using the SOAP aligner tool [27], allowing up to two base mismatches. About half (51,607,107 reads) of the total reads are mapped to the contigs, and 49,821,911 reads remain unmapped. Specifically, 11,434,981 (46.01%) reads are mapped to the contigs in the rFLJ bud; 8,202,791 (56.30%) to rFLJ flower2; 17,927,893 (56.27%) to FLJ bud; 8,943,545 (54.21%) to FLJ flower2; and 4,697,897 (36.30%) to FLJ flower1. The average contig lengths are less than 1,000 bp, but the N50 contig sizes are over 1,000 bp for all libraries.

### Gene annotation and expression analysis

We used the available public information of plant genes and genomes for annotation and performed a similarity search against the Genbank non-redundant protein database (Genbank nr) using the BLASTx algorithm [21,33] with an E-value threshold of 10^−5^ and a size threshold >100 bp. We have 119,965 contigs (66.64%) shown significant similarity to known proteins based on 45,549 unique proteins. Based on the BLAST search, 86% of the contigs show similarities in the six plant species, including *Vitis viniferaRicinus communisPopulus trichocarpaArabidopsis lyrataGlycine max*, and *Nicotiana tabacum* ( Additional file 1: Figure S3), and the fractions of sequences that match to what in *V. vinifera* are more than 50% for all five libraries. Due to the absence of genome information for FLJ, the full-length cDNA set of *V. vinifera* (RefSeq project by NCBI) served as the best reference for clustering and combining FLJ and rFLJ data. Moreover, our results indicate that the proportion of the sequences with matches in the Genbank nr database is greater among the longer contigs. For instance, we observed 98.6% matching efficiency for the sequences longer than 2,000 bp but it decreased to 50.8% when the sequence lengths dropped to 100 to 500 bp. The matching efficiencies for the sequences ranging in 500–1,000 bp, 1,000–1,500 bp, and 1,500–2,000 bp, are 90.5%, 96.6%, and 98.2%, respectively.

We defined the FLJ/rFLJ genes using LASTZ [29] and *V. vinifera* full-cDNAs (15,674  *V. vinifera* genes and E value of 10^−10^) as the reference. Fragmented genes were also identified and joined as ESTs. The FLJ/rFLJ transcriptomes were defined based on the criterion: at least one contig mapped to a reference gene. Nearly 30% of the total reference genes have matches to the FLJ/rFLJ contigs. Finally, we have 5,480, 5,310, 5,818, and 5,131 unigenes identified in rFLJ bud, rFLJ flower2, FLJ bud, and FLJ flower2, respectively. Only the FLJ flower1library (4,483 genes) has less than 5,000 unigenes identified.

### Functional analysis

We carried out functional and pathway analyses using the Kyoto Encyclopedia of Genes and Genomes (KEGG; http://www.genome.jp/kaas-bin/kaas_main?mode=est_b;22), and 180,020 sequences with significant matches were assigned to 276 KEGG pathways. Of the total, 21,692 unigenes have enzyme commission (EC) numbers ( Additional file 2: Table S3). We attempted to map major compounds that are involved in the biosynthesis of phenylalanine, terpenoid backbone, and fatty acid to the citric acid cycle, glycolysis, and sucrose metabolic pathways based on sequence homologies to the known plant genes ( Additional file 1: Figure S4). We categorized a total of 1,321 unigenes involved in the biosynthetic pathways. In addition to genes assigned to metabolic pathways, 18,417 unigenes are attributable to functions in genetic information processing, membrane transport, signal transduction, immune system, and environmental adaptation. These results demonstrated the power of high-throughput sequencing in identifying novel genes in non-model organisms, providing a valuable resource for investigating specific processes, functions, and pathways involved in active compound formation and their accumulation in FLJ flowers.

### Analysis of differentially-expressed genes (DEGs)

We calculated gene expression level based on unique read counts and RPKM values (reads per kilobase of exon model per million mapped reads) for each contig and ESTs (the length of the ESTs was the exon length for the contigs). Two methods were used to define DEGs: DEGseq software based on *p* value estimates and 2-fold RPKM differences between the two libraries as a threshold for each gene. The numbers of DEGs between different datasets are: (1) 2,316 between the FLJ bud and flower1, (2) 1,713 between the FLJ bud and flower2, (3) 1,163 between FLJ flower1 and flower2, (4) 666 between rFLJ and FLJ bud, and (5) 692 between rFLJ and FLJ flower2.

We identified 262 (26%) up-regulated and 663 (67%) down-regulated genes in the flower bud in comparison with flower1 and flower2 in FLJ (the group 1 comparison; Additional file 1: Figure S5). These DEGs are concentrated on certain pathways, such as biosynthesis of plant hormones (41 DEGs), biosynthesis of terpenoids and steroids (28 DEGs), ribosomes (25 DEGs), biosynthesis of phenylpropanoids (24 DEGs), and biosynthesis of alkaloids derived from terpenoids and polyketides (21 DEGs). Up-regulated genes are absent in certain pathways, such as fatty acid and unsaturated biosyntheses. The total number of down-regulated genes in the three fatty acid-related pathways is 12, which is higher than that found in other pathways without up-regulated genes ( Additional file 1: Figure S6).

One of the down-regulated DEGs in pathogenesis (GO:0009405) is glyceraldehyde-3-phosphate dehydrogenase (G3PD, EC:1.2.1.12; Additional file 1: Figure S7). G3PD catalyzes the conversion of glyceraldehyde-3-phosphate to 1,3-bisphosphoglycerate in glycolysis and plays a critical role in the control of plant metabolism and development [34]. Munoz-Bertomeu reported [35] that the expression and catalytic activity of G3PD in anthers are necessary for mature pollen development of Arabidopsis. Exine formation in developing pollen exhibits an intricate pattern, primarily comprised of a polymer of fatty acids and phenolic compounds [36]. We show here that the transcription level of sc_FLJ_007660 is 9.57-fold and 27.54-fold higher in the group 1 comparison.

The up-regulated DEGs are involved in transport (GO:0006810), transmembrane transporter activity (GO:0022857), and substrate-specific transporter activity (GO:0022892). One of the DEGs involved in localization (GO:0051234) is a regulator of Vps4 activity in the MVB protein pathway and related to pollen tube growth [37] ( Additional file 1: Figure S7). Rapid pollen tube growth requires a high rate of sugar metabolism to meet energetic and biosynthetic demands [38]. The transcription level of sc_FLJ_015256 is 0.35-fold and 0.43-fold in the group 1 comparison and the result suggests a distinct sucrose metabolism.

### DEGs and their related metabolic pathways

**Figure 2:**
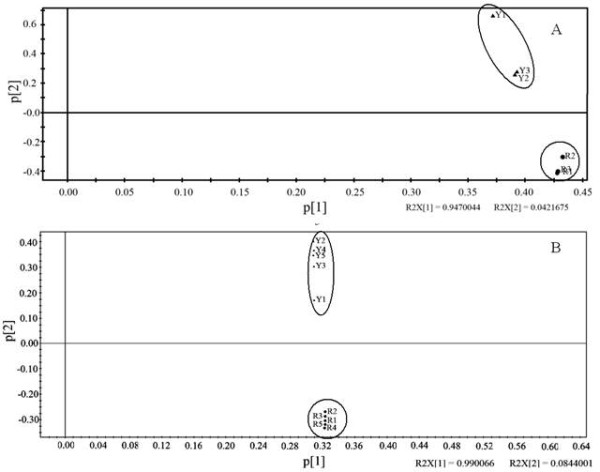
**Metabolic profile differences between flower buds of FLJ and rFLJ as detected based on PCA of GC-MS and HPLC data.** (A) PCA of metabolic profiles based on GC-MS analysis (n = 3). (B) PCA of metabolic profiles based on HPLC analysis (n = 5). Y1–Y5, FLJ samples; R1-R5, rFLJ samples. Distinct metabolic profiles that correspond to a particular species are circled in (A) and (B). PCA analyses were performed by using the SIMCA-P + (12.0.0.0.0) program (Umetrics AB, Tvistevdgen 48 Umea 907 19, Sweden). p [1] is the first principal component, and p [2] the second principal component.

We further investigated several metabolic pathways and selected several representative pathways for more detailed analyses, including phenolic acids, terpenoid and fatty acid metabolism, glycolysis, and TCA cycles (Figure 2 and Additional file 1: Figure S8).

### Phenolic acid pathway

**Figure 3:**
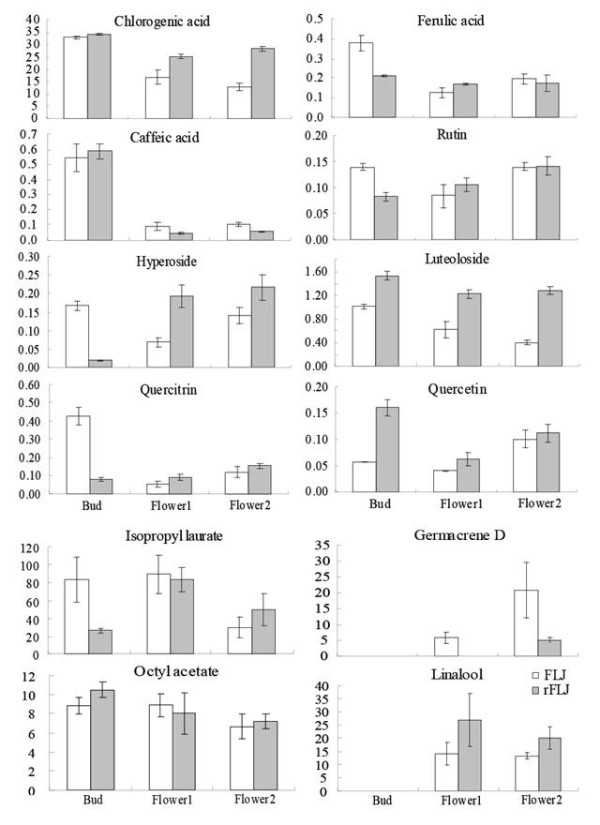
**The active compound contents in flowering samples of FLJ and rFLJ.** (A) The content of eight compounds: chlorogenic acid, caffeic acid, ferulic acid, rutin, luteoloside, hyperoside, quercitrin and quercetin (mg/g DW), as analyzed by using HPLC and calculated based on a linear formula ( Additional file 2: Table S2). Error bars (SEs; n = 5) were calculated by using Excel software. (B) The content of volatile compounds (Metabolite/pentadecanol [IS] peak area ratio/1000) in FLJ and rFLJ. The Y axis indicates the relative quantification of metabolites by normalization of their response values to pentadecanol.

Most phenolic compounds are derived from the phenylpropanoid pathway (Figure 3). L-Phenylalanine is first catalyzed to trans-cinnamic acid by phenylalanine ammonia-lyase (PAL, EC:4.3.1.24), and subsequently to p-coumaric acid by trans-cinnamate 4-hydroxylase (C4H). P-coumaric acid can be converted into caffeic acid by p-coumarate 3-hydroxylase (C3H) or to p-coumaroyl-CoA by 4-coumarate-CoA ligase (4CL, EC:6.2.1.12). The four known products of the p-coumaroyl-CoA-related reactions are as follows: (1) caffeoyl-CoA O-methyltransferase (COMT, EC:2.1.1.104) for feruloyl-CoA, (2) cinnamyl-alcohol dehydrogenase (EC:1.1.1.195) for p-coumaryl alcohol, (3) shikimate O-hydroxycinnamoyltransferase (HCT) for p-coumaroyl quinic acid and chlorogenic acid, and (4) chalcone synthase (CHS, EC:2.3.1.74) and chalcone isomerase (CHI, EC:5.5.1.6) for naringenin. In addition, naringenin can be catalyzed to form dihydrokaempferol by dihydroflavonol −4-reductase (DFR, EC:1.1.1.219), to become apiforol by flavanone 4-reductase (F4R) or a DFR homolog, or to become eriodictyol by flavonoid 3′-hydroxylase (F3′H). Eriodictyol can be further catalyzed to become luteoforol by a homolog of DFR (EC:1.1.1.219) and to be luteolin by flavones synthase (FNS). Dihydrokaempferol can also be further converted into *cis*-3,4-leucopelargonidin by flavanone 3β-hydroxylase (F3H), and eventually to quercetin and rutin by flavonol synthase (FLS).

**Figure 4:**
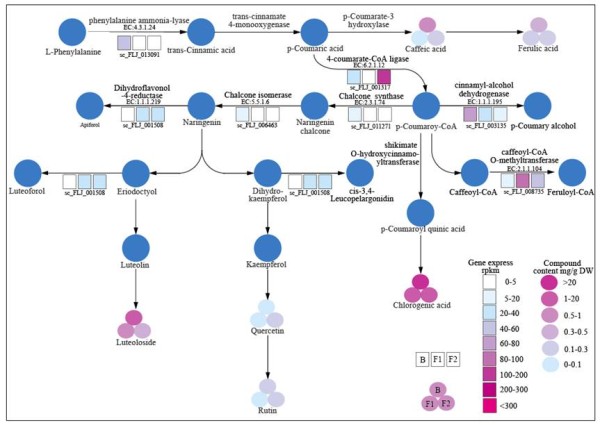
**Pathway of active compound biosynthesis in the flowering samples of FLJ.** Circles represent compounds and six different colors indicate the content of active compounds ranging from 0 to above 20 mg/g DW. Squares represent gene express level and the nine different colors indicate RPKM values of the ESTs. Three-fold circles and three-row squares display the content of compounds and gene express levels. Abbreviations: B, bud; F1, flower1; and F2, flower2.

In the group 1 comparison, we found that the transcription levels of PAL, EC:2.3.1.74, EC:5.5.1.6, and EC:1.1.1.195 are down-regulated, whereas EC:2.1.1.104 and EC:1.1.1.219 are up-regulated in FLJ buds (Figure 2). In the group 2 comparison, the transcription levels of seven DEGs (EC:1.14.13.11, EC:2.1.1.104, EC:6.2.1.12, EC:3.2.1.21, EC:4.3.1.24, EC:1.14.11.9, and EC:2.3.1.74) involved in phenylpropanoid and flavonoid biosynthesis are down-regulated. Our data strongly support the correlation between metabolic pathways and their related gene expressions (Figure 4).

### Terpenoid pathway

In the group 1 comparison, we obtained five DEGs in terpenoid backbone biosynthesis. The transcription levels of 3-hydroxy-3-methylglutaryl-CoA reductase (HMGR, [EC:1.1.1.34]) in the mevalonate pathway, and 4-diphosphocytidyl-2-C-methyl-D-erythritol kinase [EC:2.7.1.148], isopentenyl-diphosphate delta-isomerase [EC:5.3.3.2], (E)-4-hydroxy-3-methylbut-2-enyl-diphosphate synthase [EC:1.17.7.1], and 4-hydroxy-3-methylbut-2-enyl diphosphate reductase [EC:1.17.1.2] in the plastid MEP/DXP pathway are all down-regulated. Furthermore, the transcription levels of six DEGs (EC:1.1.1.34, EC:2.7.1.148, EC:2.2.1.7, EC:5.3.3.2, EC:1.17.1.2) are down-regulated but farnesyl diphosphate synthase [EC:2.5.1.1 2.5.1.10] is up-regulated. The combination of the different trends results in an increase in germacrene D content in FLJ buds.

### Fatty acid pathway

The key enzymes involved in fatty acid metabolism, aldehyde dehydrogenase [EC:1.2.1.3,1 1.2.1.8], acyl-CoA oxidase [EC:1.3.3.6], and long-chain acyl-CoA synthetase [EC:6.2.1.3], are all down-regulated in the group 1 comparison. Other DEGs, such as 3R-hydroxymyristoyl ACP dehydrase [EC:4.2.1.-], acyl-ACP desaturase [EC:1.14.19.2], fatty acyl-ACP thioesterase B [EC:3.1.2.14 3.1.2.-], acyl-CoA oxidase [EC:1.3.3.6], and omega-3 fatty acid desaturase (delta-15 desaturase) [EC:1.14.19.-], involved in the biosynthesis of fatty acids and unsaturated fatty acids, are also down-regulated. In addition, five DEGs (EC:1.14.19.-, EC:1.3.1.-, EC:3.1.2.14/3.1.2.-) involved in fatty acid and unsaturated fatty acid biosynthesis are also down-regulated in the group 2 comparison. Our expression data again are in complete agreement with the metabolic data (Figure 4).

### Primary/central metabolism

Our group 1 comparison results lead to the identifications of down-regulated DEGs: eight (EC:1.1.1.37, EC:1.2.4.1, EC:1.3.99.1, EC:1.8.1.4, EC:2.3.3.1, EC:2.3.3.8, EC:6.2.1.4/6.2.1.5) in the citrate cycle, eleven (EC:1.1.1.1, EC:1.2.1.12, EC:1.2.4.1, EC:1.8.1.4, EC:4.2.1.11, EC:5.3.1.1, EC:5.4.2.2, EC:1.2.1.31/1.2.1.8) in glycolysis/gluconeogenesis, and five (EC:1.1.1.22, EC:2.4.1.13, EC:2.7.7.27, EC:3.2.1.2 and EC:5.4.2.2) in starch and sucrose metabolism. In addition, we found five up-regulated genes in the group 1 comparison: succinate dehydrogenase (ubiquinone) iron-sulfur protein [EC:1.3.5.1], pyruvate kinase [EC:2.7.1.40], α, α-trehalose-phosphate synthase (UDP-forming) [EC:2.4.1.15], 4-α-glucanotransferase [EC:2.4.1.25], and beta-amylase [EC:3.2.1.2]. In the group 2 comparison, we defined five down-regulated genes (EC:1.1.1.37, EC:1.8.1.4, EC:2.3.3.8, EC:4.1.2.13, EC:3.2.1.2, and EC:3.2.1.21) and they are involved in the citrate cycle, glycolysis/gluconeogenesis, and starch and sucrose metabolism. In contrast, we have eight up-regulated genes: EC:1.8.1.4, EC:4.2.1.11, EC:5.1.3.3, EC:5.4.2.2, EC:6.2.1.1, EC:1.1.1.22, EC:2.7.7.27, EC:5.4.2.2, and EC:2.7.1.40.

We also validated some less-abundant DEGs as representatives for most of the above-mentioned pathways using qRT-PCRs, and the results are consistent with the RNA-seq data ( Additional file 2: Table S5).

### Integrated analysis of active compound abundance and gene expression

**Figure 5:**
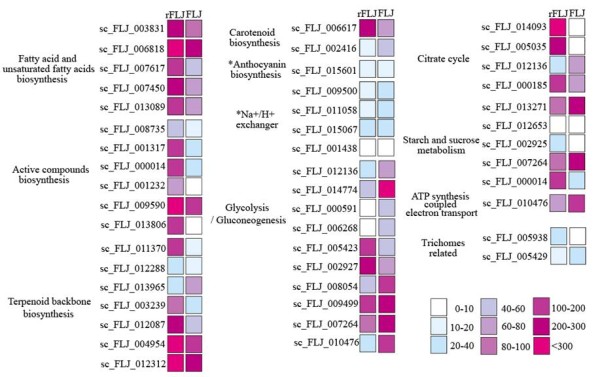
**Gene transcription level in flower buds of FLJ and rFLJ.** The square represents gene express levels, and the nine colors indicate the RPKM values of the ESTs as calculated according to the grape full-length cDNA sequences. * denotes no differential expression between FLJ and rFLJ.

According to a previous report, the content of chlorogenic acid in the FLJ flower bud is higher than that of the flowers [10]. Our study shows that the contents of chlorogenic acid and four other active compounds (caffeic acid, ferulic acid, luteoloside, and quercitrin) are actually lower, aside from three others (isopropyl laurate, linalool and germacrene D) that remain unchanged in the three different flowering stages (Figure 5A). We also found that related enzymes PAL, CHS, and CHI are up-regulated in the group 1 comparison (Figure 3). These results indicate that there are reduced expressions of the key genes involved in phenolic compound skeleton biosynthesis, leading to changes in the FLJ active compounds during flowering, which provide additional information for the medicinal value of the flower buds. We further found that the content of isopropyl laurate has a similar trend as what we observed in fatty acid biosynthesis.

The MEP/DXP and MVA pathways are thought to be interdependent during the biosynthesis of isoprenoids [39]. Sesquiterpene is synthesized by the MVA pathway during the isoprenoid biosynthetic system in plants and the HMGR multi-gene family catalyzes the synthesis of mevalonate, which is a precursor in this pathway [40]. The inducible HMGR enzyme activity is required for sesquiterpene accumulation in tobacco cell suspension cultures [41]. The transcription level of HMGR has no effect and farnesyl diphosphate sythase (FPS) is only transiently induced after emission of (−)-germacrene D in poplar [42]. However, our results suggest that the content of sesquiterpene may be negatively controlled by HMGR and the up-regulation of FPS may increase the production of germacrene D.

### Analysis of active compounds in FLJ and rFLJ

To gain additional insight into the active compounds and metabolic pathway maps in FLJ and rFLJ, we generated metabolic profiles of the active compounds from different tissues using HPLC (Figure 5) and observed reduced production of chlorogenic acid, caffeic acid, ferulic acid, luteoloside, and quercitrin after flowering (Figure 5A). Our GC-MS analysis also indicates variable fatty acid and terpenoid contents in the flowering stages (Table 2). In addition, we observed the decreased content of isopropyl laurate and the increased contents of linalool and germacrene D in the flowering process (Figure 5B). Isopropyl laurate, linalool, and germacrene D are all known major volatile chemicals released in full-bloomed flowers [43].

**Table 2:** GC-MS analysis of volatile compounds

Peak No.	RT	rFLJ			FLJ			Compounds
		B	F1	F2	B	F1	F2	
7	9.767			*				cis-3-hexenyl acetate
8	10.767	*	*	*	*	*	*	2-Ethylhexanol
10	13.908		*	*		*	*	Linalool
12	16.092	*	*	*	*	*	*	Octyl acetate
17	31.292			*		*	*	Germacrene-D
19	40.925	*	*	*	*	*	*	Isopropyl laurate
22	44.417			*				Farnesol
26	45.956	*	*	*	*	*	*	Pentadecanol (IS)
28	46.950			*	*			1-Octadecyne
30	47.450	*	*	*	*	*	*	Isobutyl O-phthalate
31	48.117	*	*		*	*		2-Nonadecanone
34	48.917	*	*	*	*	*	*	Methyl glycol phthalate
35	51.217			*				Nerolidol acetate
36	51.617			*			*	Farnesol isomer
39	52.717	*						Normal-docosane
40	55.2 00	*	*	*		*	*	Didecyl ether
41	55.7 00	*	*	*		*	*	Docosanoic acid, methyl ester
44	56.717		*	*		*	*	Allyl stearate
45	58.867	*	*	*	*	*	*	Heptacosane
46	58.950			*			*	Arachic alcohol
47	59.65 0	*	*	*	*	*	*	Tetracosanoic acid, methyl ester
52	64.692	*	*	*	*	*	*	Nonacosane

*Abbreviations:* FLJ, *Lonicera japonica* Thunb; rFLJ, *Lonicera japonica* Thunb. var. chinensis (Watts.); B, bud; F1, flower1; F2, flower2; RT, retention time; *, present in sample; and IS, internal standard.

**Figure 6:**
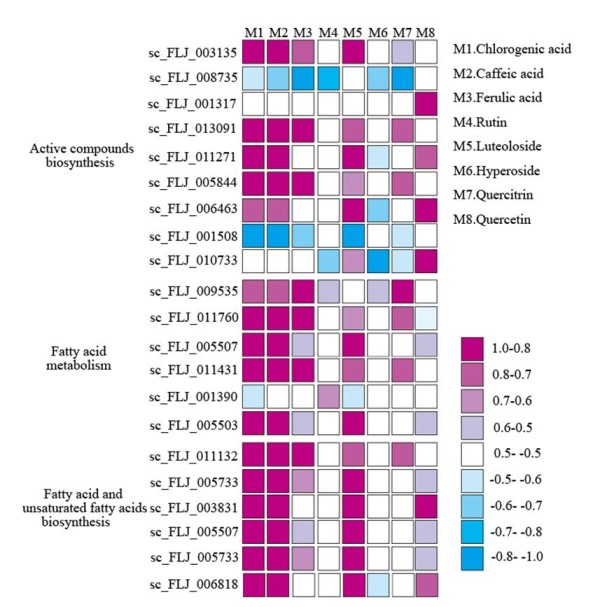
**Correlation between gene express level and active compound accumulation in FLJ.** ESTs numbers and metabolites are shown in the key. M1–M8 are active compounds. The color key provides R values for the correlations calculated for the flower development datasets. The RPKM values of ESTs were calculated according to the grape full-length cDNA sequences.

We also carried out PCA analysis on selected organic compounds; phenolic acids, fatty acids, and terpenoids are all detected by using GC-MS and HPLC. These compounds form two independent groups (Figure 6), where the contents of chlorogenic acid, luteoloside, quercitin, and isopropyl laurate are higher overall in the rFLJ flower buds as compared to those of FLJ but the germacrene D content is lower in rFLJ.

### Interactions of secondary and primary/central metabolisms

The production of secondary metabolites is tightly associated with pathways of primary/central metabolism, such as glycolysis, the shikimate pathway, the production of aromatic amino acids, and aliphatic amino acids [44]. GC-MS and HPLC analysis detected three compound groups produced from primary metabolisms in FLJ and rFLJ: phenolic acids, terpenoids, and fatty acids ( Additional file 1: Figure S4).

### Phosphoenolpyruvate metabolism

Phosphoenolpyruvate (PEP) is an example of glycolysis intermediates. It is indispensable for energy metabolism in the cytosol and delivers ATP and pyruvate catalyzed by cytosolic pyruvate kinase (PK) [45]. Inside the plastids, PEP acts as a precursor for at least four metabolic pathways: fatty acids, isoprenoids, branched chain amino acids, and the shikimate pathway [46]. However, chloroplasts and most non-green plastids lack the ability to produce PEP *via* glycolysis, because their enolase is either absent or has low activity [47]. In this study, we found that enolase (sc_FLJ_010870) transcription is 1232.8-fold higher in the FLJ flower buds when compared with flower1, and no detectable expression of the enzyme in flower2. Therefore, the formation of PEP is negatively correlated with FLJ flowering. In contrast, the transcription level of PK (sc_FLJ_000591), a competing enzyme, is 0.11-fold and 0.54-fold in the group 1 comparison. We neither observed significant changes in the flavonoid content nor in ABA and fatty acids contents as what was reported in an eno1 mutant when compared with the wild type of *A. thaliana*[46]. We did found that the transcription level of enolase is up-regulated in buds as compared with the two flower stages, as well as in the group 2 comparison, but showed no correlation with the phenolic acid content. In contrast, the transcription level of PK is higher in the same comparison. Our combined analysis of biosynthesis of phenolic acid, terpenoids, and fatty acids did indicate that there is a potential correlation between PK expression level and the contents of flavonoids, terpenoids, and fatty acids in FLJ.

### ATP function

ATP synthesis could also have influences on the interaction of primary and secondary metabolisms. We assessed the predicted phosphorylation sites of the relevant enzymes ( Additional file 2: Table S4) and found that all are candidates for phosphorylation-based regulation. Of the defined DEGs, a study in animals showed that HMGR exists in both active (dephosphorylated) and inactive (phosphorylated) forms [48]. PK has also been shown to be more susceptible to inhibition by ATP [49]. We analyzed the differential expression of ATP synthesis-coupled electron transporters and found that this gene family is down-regulated in the group 1 comparison and up-regulated in the group 2 comparison. Prediction of phosphorylation sites from protein sequences indicates that HMGR (sc_FLJ_012288) has nine phosphorylation sites, whereas pyruvate kinase (sc_FLJ_000591) has four phosphorylation sites.

### Sugar regulation

Previous studies on primary and secondary metabolisms have indicated that they are linked *via* the phenylalanine pool [50,51]. Therefore, it is possible that a reduced carbon flux in the phenylpropanoid pathway may affect carbohydrate metabolism.

As Matt et al. [52] showed that an increase in the sugar/amino acid ratio resulted in an elevated production of carbon-rich phenylpropanoids, we found that biosynthesis of phenylpropanoids is down-regulated in both group 1 and group 2 comparisons, and the transcription level of β-glucosidase that generates β-D-glucose and α-D-glucose is down-regulated in the group 2 comparison. Furthermore, sucrose synthase that creates sucrose is down-regulated in the group 1 comparison but β-amylase that generates maltose is up-regulated in the group 1 comparison and down-regulated in the group 2 comparison.

Moriizumi [53] reported that glucose-regulated transcription of pyruvate kinase is mediated by its glucose response element; the carbohydrate response elements are composed of two E box-like motifs separated by 5 bp and is recognized by two basic helix-loop-helix/leucine zipper (bHLH/LZ) proteins [54,55]. In the group 2 context, down-regulation of pyruvate kinase may be mediated by down-regulating glucose biosynthesis, and in the group 1 context, however, the transcription level of glucose biosynthetic enzymes is insignificant albeit up-regulated pyruvate kinase. Finally, two bHLH (sc_FLJ_008421 and sc_FLJ_006390) proteins are seen up-regulated and down-regulated in the group 1 and 2 comparisons, respectively.

A putative sequence for a carbohydrate-response-element binding protein (sc_FLJ_004075) is obtained based on sequence homology (a homolog in Norway rat; EMBLCDS:BAB77523 and in chicken GenBank: ABV72703.1). The transcript is up-regulated in the group 1 comparison, suggesting that bHLH transcription factors may be involved in regulating the response of PK to glucose in FLJ.

### The complexity of defining orthologs and paralogs for key metabolic pathways

Since gene duplication is very common in plant genomes, we made an effort to differentiate orthologs and paralogs from all homologs. The overall sequence identity between FLJ and rFLJ contigs is 99.0%. In our analysis, we selected 55 DEGs sequences from all pathway-related genes, where six of the selected genes have slightly lower identities, about 97.4% ( Additional file 2: Table S6).

**Figure 7:**
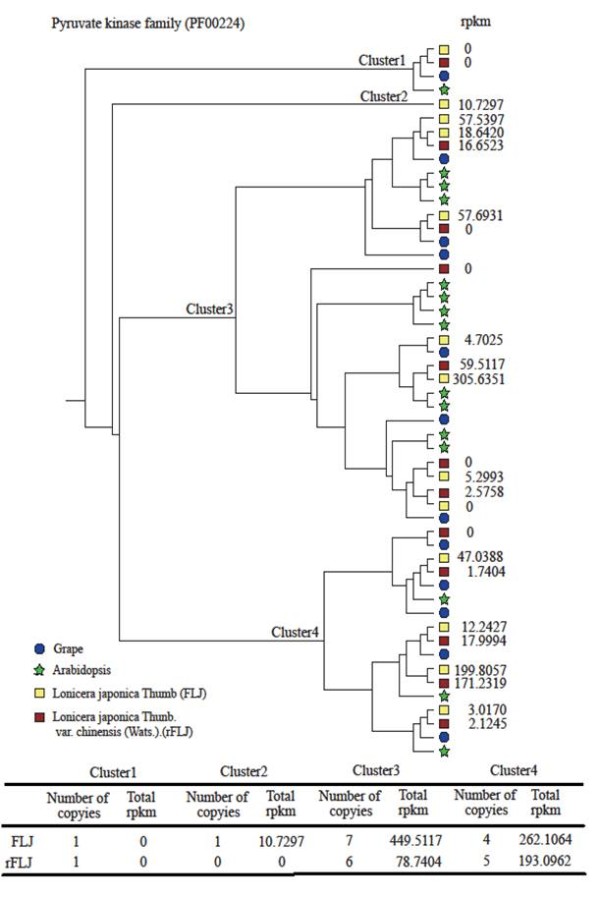
**Phylogeny of predicted amino acid sequences and expression of the pyruvate kinase homologs between the FLJ and rFLJ flower buds and the pyruvate kinase family genes in Arabidopsis and grape.** The phylogenetic tree was constructed based on the neighbor-joining method (ClustalW2). Pyruvate kinase homologs were identified based on the unique domain (PF00224) in the PFAM database.

We identified the orthologs and paralogs of PAL, CHS, HMGR, and PK based on the genome sequences of *Arabidopsis* and grape (http://www.phytozome.net; Additional file 2: Table S7) and built phylogenetic trees (Figure 7 and Additional file 1: Figure S9). First, between FLJ and rFLJ, the PAL family genes clustered into two groups; one contains a pair of orthologs that have no detectable expression in the flower buds and another has paralogs expressed at high levels. The total RPKM of PAL paralogs is 5.9-fold higher in rFLJ when compared to FLJ. Second, we have two pairs of CHS orthologs in both FLJ and rFLJ; the FLJ paralogs expressed at high levels, 17-fold higher than those in rFLJ. Third, in FLJ and rFLJ, HMGR genes are also clustered into two groups and each has two pairs of orthologs. The FLJ paralogs are expressed at low levels to the extent that they may become pseudogenes already. The total RPKM of HMGR paralogs is 1.6-fold higher in rFLJ than in FLJ. Finally, there are four groups of PK genes and the expressed PK paralogs primarily present in two of the four clusters (Cluster 3 and Cluster 4; Figure 7).

## Discussions

### Gene expression data provide more comprehensive understanding of FLJ as medicinal plant

Although several studies have reported that the content of chlorogenic acid is higher in the flower buds as compared to that in flowers, it has long been disputed as to which organ has the highest medical value due to the applications of controversial evaluation methods. In this study, we obtained a sufficient amount of transcriptomic data from both young buds and mature flowers, and carried out an integrated analysis on the variations of gene expression and the contents of active compounds. Our data from different flowering stages indicate that the contents of eight major active compounds either decreased (five of them) or remain unchanged (three of them) and that the biosynthesis of the active compounds is overall higher in the buds than in the flowers. In addition, many key enzymes, such as PAL, CHS, and CHI, are up-regulated in the FLJ flower buds (Figure 4). PAL is a key enzyme in the synthesis of phenylpropanoid phytoalexins and other phenolics [56]. Previous data have shown that the PAL expression levels decline during flowering in *Nicotiana*[57], and the decrease enzyme activity corresponds to the decreased major phenylpropanoid compounds. Our data add further support for this notion, as we found that the CHS and CHI genes are also significantly down-regulated in the later flowering stages. CHS functions to produce flavonoid precursors, and CHI plays a major part in the cyclization reaction from chalcone to flavanone [58].

The quality of herbal medicine has been very difficult to control and to evaluate primarily because of the complexity and incomplete knowledge of the active medicinal compounds. The primary methods that have been used for quality evaluation of Chinese herbal medicines are chemical and pharmacological analyses. Chemical evaluations showed that chlorogenic acid and luteoloside are two common active compounds found in FLJ. However, content and fingerprint analysis of one or more of these compounds are not indicative for the medicinal value of the plant, and our genomic approach provides a comprehensive survey. Our study generated gene expression data for terpenoids and fatty acid biosynthesis and increased valuable knowledge on other FLJ compounds.

Both fatty and phenolic acid biosyntheses may depend on the interaction between the two pathways [59,60]. In FLJ, the transcription level of long-chain acyl-CoA synthetase (sc_FLJ_011431) and 4-coumarate-CoA ligase (sc_FLJ_001317) decreases after flowering. Since the contents of phenolic compounds and fatty acids are down-regulated in both the group 1 and 2 comparisons, some of the biosynthetic genes for phenypropanoid compounds and fatty acids may be regulated in a similar way or function as similar enzymes (such as sharing catalytic domains).

Changes in phenolic acids, fatty acid biosynthesis, and the MEP/DXP pathway show a decreased trend in gene expression from the medicinal organ (buds) to the non-medicinal organs (flower1 and flower2). In addition, we used phylogenetic tools for paralog analysis and revealed that the mRNA levels of these enzymes have higher expressions in the flower buds of both FLJ and rFLJ. We found that a total of eighteen enzymes in these biosynthetic networks are differentially expressed ( Additional file 2: Table S9).

### Enzymatic divergence of orthologs and paralogs results in gene function variation and active compound content

Secondary metabolism varies intensively, even between two closely related taxonomic groups, and the underlining functional variations, such as enzyme activities, often lead to the production of unique compounds. In this study, we identified several orthologous enzymes as well as their related paralogs and evaluated their evolutionary relatedness between FLJ and rFLJ. We observed that the RPKM values of PAL, CHS, and HMGR are higher in rFLJ than FLJ, and these variations in expression may lead to alternations in the active compound contents between in the two plants.

In contrast to specific evolutionary changes to individual enzymes during speciation, most of the functional variations appear to be related to gene or genome duplications [61]. After gene duplication events, most paralogs are lost over time, but those have survived often gain new functions (neo-functionalization), partition the original function into different time and tissues (subfunctionalization), or have lost their functions (nonfunctionalization). Our data indicate that gene duplication in FLJ may provide an opportunity for neo-functionalization, whereby the PK gene and its orthologs and paralogs may evolve to have complementary enzyme functions. For instance, an FLJ PK paralog turns out to have a high RPKM value and may undergo neofunctionalization, whereas the expression of another paralog in FLJ is higher than that of rFLJ. There is a third PK paralog whose expression levels are quite similar in both plants. Nevertheless, the divergence of gene expression due to duplicated genes appears to play direct roles in the production of active compounds in FLJ and rFLJ.

## Conclusion

**Figure 8:**
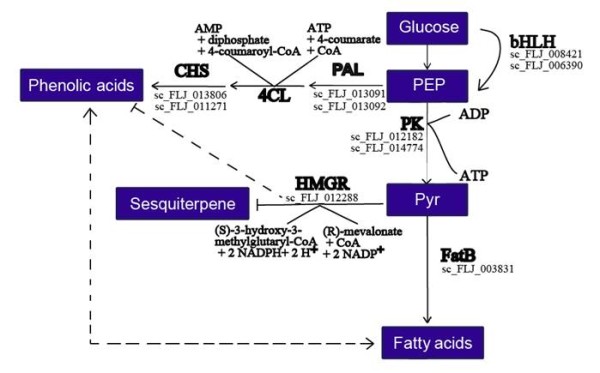
**Key enzymes and proteins in regulating biosynthesis of phenolic acids in FLJ.** Phenolic acids are produced from PEP by PAL, 4CL, and CHS. PK, 4CL, and HMGR are regulated by ATP. HMGR is related to phenolic acid and hyperoside. The biosynthesis of phenolic acids is coordinated closely with fatty acids. Converting glucose to PEP is regulated by bHLH. Abbreviations: CHS, chalcone synthase; 4CL, 4-coumarate-CoA ligase; PAL, phenylalanine ammonia-lyase; PK, pyruvate kinase; HMGR, 3-hydroxy-3-methylglutaryl-CoA reductase; FatB, fatty acyl-ACP thioesterase B; PEP, phosphoenolpyruvate; and Pyr, pyruvate.

We used a comparative approach to address whether transcriptomes can be informative for the analysis of active medicinal compounds in herbal plants. Our study not only provided an initial description of the expression profiles of FLJ flowers, but also identified the enzyme pool that can be used to evaluate FLJ quality in future studies. We also associated metabolic pathways involved in processing active medicinal compounds to the expressions of their catalytic enzymes. We also used sequence evolution as a tool to identify orthologs and paralogs, as well as pathways for the biosynthesis of phenolic acid and its interactions with other pathways (Figure 8), and revealed that functional divergence of orthologs and paralogs may lead to variations in gene functions that control the active compound contents among different tissues and plants.

## Abbreviations

FLJ, Lonicera japonica Thunb; rFLJ, Lonicera japonica Thunb. var. chinensis (Wats.); qRT-PCR, Quantitative reverse transcriptase PCR; RT-PCR, Reverse transcriptase PCR; RNA-seq, RNA sequencing.

## Competing interests

The authors declare that they have no competing interests.

## Authors’ contributions

JY and LQH designed the study. YY and SSQ collected samples and did GC-MS analysis. YY, MHL, and XW did HPLC analysis. LPS, GML, and YY carried out data analysis. XMW contributed to RNA-seq library construction and sequencing. JY and YY contributed writing of the manuscript. All authors read and approved the final manuscript.

## Supplementary Material

Additional file 1 **Figure S1. Comparing volatile compound of FLJ and rFLJ using Gas chromatography-mass spectrometry.** A, Bud, B, flower1, C, flower2. Black line represent FLJ and Red line represent rFLJ. FLJ, *Lonicera japonica* Thumb; rFLJ, *Lonicera japonica* Thunb. var. chinensis (Wats.). **Figure S2. HPLC analysis of active compounds in FLJ and rFLJ**. A, Mix of standard compounds. Chlorogenic acid (RT, 13.20; MW, 354.31); Caffeic acid (RT, 17.39; MW, 180.15); ferulic acid (RT, 24.24; MW, 194.18); Rutin (RT, 24.24; MW, 610.52); Luteoloside(RT, 25.27; MW,448.4); Hyperoside (RT, 25.35; MW, 464.37); Quercitrin (RT, 28.65; MW, 448.38); Quercetin (RT, 38.04; MW, 302.24). B, buds of rFLJ; C, buds of FLJ. FLJ, *Lonicera japonica* Thumb; rFLJ, *Lonicera japonica* Thunb. var. chinensis (Wats.). **Figure S3. BlastX analysis result of contigs in FLJ and rFLJ with all non-redundant (NR) database in Genebank**. Six species,Vitis vinifera, Ricinus communis, Populus trichocarpa, Glycine max, Arabidopsis lyrata, Nicotiana tabacum has highest identity with FLJ and rFLJ bud. E-value cut-off was lower than 1e^-5^. FLJ, *Lonicera japonica* Thumb; rFLJ, *Lonicera japonica* Thunb. var. chinensis (Wats.). **Figure S4. Pathways of major chemical compounds in*Lonicera japonica*Thumb (FLJ)**. All of contigs from three FLJ libraries were annotated with KEGG database. The six pathways, phenylalanine metabolism, terpenoid backbone, fatty acid biosynthesis, citric acid cycle, glycolysis and sucrose metabolism were analysis. Green rectangles repress enzymes finding in FLJ transcriptome. **Figure S5. Analysis of gene differential express using MA-plot-based method.** M is the Y -axis and represents the intensity ratio, and A is the X-axis and represents the average intensity for each transcript. The red points are the genes identity as differentially expressed. FLJ, *Lonicera japonica* Thumb; rFLJ, *Lonicera japonica* Thunb. var. chinensis (Wats.). **Figure S6 Pathway assignment based on KEGG analysis of differential express genes between buds and other two flower developmental periods of*Lonicera japonica*Thumb (Group1)**. A, Number of contig with down-regulated and up-regulated differential express genes. B, Number of contig with only up-regulated differential express genes. **Figure S7. Gene Ontology classification of differential express genes.** The results are summarized in three main categories: Biological process, Cellular component and Molecular function. FLJ, *Lonicera japonica* Thumb; rFLJ, *Lonicera japonica* Thunb. var. chinensis (Wats.). **A**, Gene Ontology classification of differential express genes between buds and two other flower developmental period of FLJ. Bud and flower1, differential express genes between bud and flower1; Bud and flower2, differential express genes between bud and flower2; up-regulated, up-regulated express genes in both in between bud and flower1 and in between bud and flower2; down-regulated, down-regulated express genes in both in between bud and flower1 and in between bud and flower2. **B**, Gene Ontology classification of differential express genes between buds of FLJ and rFLJ.Group2, differential express genes between buds of FLJ and rFLJ. up-regulated, up-regulated express genes; down-regulated, down-regulated express genes. **Figure S8. Gene express level in bud, flower1 and flower2 of*Lonicera japonica*** Thumb. Square represents gene express level and nine kinds of color indicate rpkm of scaffolds. B, bud;F1, flower1; F2, flower2. rpkm of scaffolds was calculated according to Grape full-length cDNA sequence. **Figure S9. Phylogenetic analysis of the predicted amino acids sequences and expression level of the Phenylalanine ammonia-lyase (PAL), 3-hydroxy-3-methylglutaryl-CoA reductase (HMGR) and chalcone synthase (CHS) homologues among the buds of FLJ and rFLJ and PAL,HMGR and CHS family genes in Arabidopsis and Grape.** The phylogenetic tree was constructed by the neighborjoining method using ClustalW2. Identification of PAL,HMGR and CHS homologues was by searching the domain(PF00221, PF00368 and PF00195, respectively) in PFAM database. FLJ, Lonicera japonica Thumb; rFLJ, Lonicera japonica Thunb. var. chinensis (Wats). Click here for file

Additional file 2 **Table S1. The Elution Conditions of HPLC Analysis.** Note: T, Retention time; A, mobile phase deionized water- formic acid (99:1, v/v); B, mobile phase methanol. **Table S2. Formula of Active Compound Content.** Note: Calibration plots of eight standards were constructed on the basis of peak areas (y) using seven different concentration solutions (x). All plots were linear in the examined ranges, and linear ranges had been shown as the concentration of the standard compounds (μg mL-1). The r referred to the correlation coefficient of the equation. The standard compounds Chlorogenic acid (110753), Caffeic acid(110885), ferulic acid(110773), Rutin(100080), Luteoloside(111720), Hyperoside(111521), Quercitrin(111538) and Quercetin (100081) were purchased from National institutes for food and drug control, China. **Table S3. Number of Contigs in KEGG Pathways.** Note: FLJ, *Lonicera japonica* Thumb; rFLJ, *Lonicera japonica* Thunb. var. chinensis (Wats.); B, bud; F1,flower1; F2, flower2. **Table S4. Predicted Phosphorylated Sites in 34 Protein Sequence from Differential Express Gene.** Note: Predicted Phosphorylated Sites using software online (http://kinasephos2.mbc.nctu.edu.tw/) and protein sequence was perdicted by ORF finder (http://www.ncbi.nlm.nih.gov/gorf/gorf.html). **Table S5. qRT-PCR and RNA-seq Analysis of Gene Express between Buds of*Lonicera japonica*Thunb. var. chinensis (Wats.) and*Lonicera japonica*Thumb.** Note: RR/YR, the ratio of transcripted level in buds of *Lonicera japonica* Thunb. var. chinensis (Wats.) and *Lonicera japonica* Thumb. **Table S6. Orthologs Identity of Differential Express Genes Sequence between FLJ and rFLJ.** Note: FLJ, *Lonicera japonica* Thumb; rFLJ, *Lonicera japonica* Thunb. var. chinensis (Wats.) **Table S7 PAL, CHS, HMGR and PK Gene Families in Arabidopsis and Grape. Table S8. Putative Enzyme Pool to Control the Active Compounds in Buds of*Lonicera japonica*Thumb.** Click here for file

## References

[B1] Hong SZJ Yang G Luo X Earliest report of Flos Lonicerae Japonicae and its medical organ Zhongyaocai 1997 20 2

[B2] Xiang T Xiong QB Ketut AI Tezuka Y Nagaoka T Wu LJ Kadota S Studies on the hepatocyte protective activity and the structure-activity relationships of quinic acid and caffeic acid derivatives from the flower buds of Lonicera bournei Planta Med 2001 67 322 325 10.1055/s-2001-14337 11458447

[B3] Yoo HJ Kang HJ Song YS Park EH Lim CJ Anti-angiogenic, antinociceptive and anti-inflammatory activities of Lonicera japonica extract J Pharm Pharmacol 2008 60 779 786 18498715 10.1211/jpp.60.6.0014

[B4] Hsu SL Chen CY Peng WH Wu LC Wu CC Luteolin Ameliorates Experimental Lung Fibrosis Both in Vivo and in Vitro: Implications for Therapy of Lung Fibrosis J Agr Food Chem 2010 58 11653 11661 10.1021/jf1031668 20958047

[B5] Li P Qi LW Chen CY Structural characterization and identification of iridoid glycosides, saponins, phenolic acids and flavonoids in Flos Lonicerae Japonicae by a fast liquid chromatography method with diode-array detection and time-of-flight mass spectrometry Rapid Commun Mass Sp 2009 23 3227 3242 10.1002/rcm.4245 19725056

[B6] Chung JH Ryu KH Rhee HI Kim JH Yoo H Lee BY Um KA Kim K Noh JY Lim KM Anti-Inflammatory and Analgesic Activities of SKLJI, a Highly Purified and Injectable Herbal Extract of Lonicera japonica Biosci Biotech Bioch 2010 74 2022 2028 10.1271/bbb.100279 20944425

[B7] Chang KC Jeong JJ Ha YM Jin YC Lee EJ Kim JS Kim HJ Seo HG Lee JH Kang SS Rutin from Lonicera japonica inhibits myocardial ischemia/reperfusion-induced apoptosis in vivo and protects H9c2 cells against hydrogen peroxide-mediated injury via ERK1/2 and PI3K/Akt signals in vitro Food Chem Toxicol 2009 47 1569 1576 10.1016/j.fct.2009.03.044 19362115

[B8] Ge B LX Yi K Tian Y The Active Constituent and Pharmaceutical Action of Flos Lonicerae and Its Application Chinese Wild Plant Resources 2004 23 5

[B9] El-Sayed AM Mitchell VJ McLaren GF Manning LM Bunn B Suckling DM Attraction of New Zealand Flower Thrips, Thrips obscuratus, to cis-Jasmone, a Volatile Identified from Japanese Honeysuckle Flowers J Chem Ecol 2009 35 656 663 10.1007/s10886-009-9619-3 19444522

[B10] Geng S Ning X Wu H Lin H Zhao S Xu H The Structure of Flower in Different Developmental Stages in Relation to the Varieties of Chlorogenic Acid Content in Lonicera confusa Acta Botanica Yunnanica 2005 27 3 279 287

[B11] Bai GB Peng XX Li WD Wang WQ Cloning and Characterization of a cDNA Coding a Hydroxycinnamoyl-CoA Quinate Hydroxycinnamoyl Transferase Involved in Chlorogenic Acid Biosynthesis in Lonicera japonica Planta Med 2010 76 1921 1926 10.1055/s-0030-1250020 20539970

[B12] Jiang K Pi Y Huang Z Hou R Zhang Z Lin J Sun X Tang K Molecular cloning and mRNA expression profiling of the first specific jasmonate biosynthetic pathway gene allene oxide synthase from Hyoscyamus niger Russ J Genet 2009 45 430 439 10.1134/S1022795409040073 19507702

[B13] Yang M HF Zhang L Comparison on the Chlorogenic Acid Contents in Different Kinds and Different Parts of Flos Lonicerae Journal of Instrumental Analysis 2006 25 122 123

[B14] HC Lonicerae Japonicae 1998 Science Press, Beijing

[B15] Qin S YY Hu G Chen X Li X Comparison of active compounds between Lonicera japonica Thunb and their variation China J of Exper Tran Med Form 2010 16 2

[B16] Lei Z ZR Zeng R He Y Comparative experiments on antipyretic effect between Lonicera macrathodes Hands Mazz. and the certified Flos Lonicera Journal of Hunan Traditional Chinese Medicine University of Hunan 2005 5 2

[B17] Bahler J Wilhelm BT Marguerat S Watt S Schubert F Wood V Goodhead I Penkett CJ Rogers J Dynamic repertoire of a eukaryotic transcriptome surveyed at single-nucleotide resolution Nature 2008 453 U1239 U1239 10.1038/nature07002 18488015

[B18] Snyder M Nagalakshmi U Wang Z Waern K Shou C Raha D Gerstein M The transcriptional landscape of the yeast genome defined by RNA sequencing Science 2008 320 1344 1349 10.1126/science.1158441 18451266 PMC2951732

[B19] Wold B Mortazavi A Williams BA Mccue K Schaeffer L Mapping and quantifying mammalian transcriptomes by RNA-Seq Nat Methods 2008 5 621 628 10.1038/nmeth.1226 18516045 PMC13303166

[B20] Simpson JT Wong K Jackman SD Schein JE Jones SJ Birol I ABySS: a parallel assembler for short read sequence data Genome Res 2009 19 1117 1123 10.1101/gr.089532.108 19251739 PMC2694472

[B21] Altschul SF Madden TL Schaffer AA Zhang JH Zhang Z Miller W Lipman DJ Gapped BLAST and PSI-BLAST: a new generation of protein database search programs Nucleic Acids Res 1997 25 3389 3402 10.1093/nar/25.17.3389 9254694 PMC146917

[B22] Kanehisa M Goto S KEGG: kyoto encyclopedia of genes and genomes Nucleic Acids Res 2000 28 27 30 10.1093/nar/28.1.27 10592173 PMC102409

[B23] Tatusov RL Fedorova ND Jackson JD Jacobs AR Kiryutin B Koonin EV Krylov DM Mazumder R Mekhedov SL Nikolskaya AN The COG database: an updated version includes eukaryotes BMC Bioinforma 2003 4 41 10.1186/1471-2105-4-41 PMC222959 12969510

[B24] Hunter S Apweiler R Attwood TK Bairoch A Bateman A Binns D Bork P Das U Daugherty L Duquenne L InterPro: the integrative protein signature database Nucleic Acids Res 2009 37 D211 D215 10.1093/nar/gkn785 18940856 PMC2686546

[B25] Aparicio G Gotz S Conesa A Segrelles D Blanquer I Garcia JM Hernandez V Robles M Talon M Blast2GO goes Grid: Developing a Grid-Enabled Prototype for Functional Genomics Analysis St Heal T 2006 120 194 204 16823138

[B26] Maere S Heymans K Kuiper M BiNGO: a Cytoscape plugin to assess overrepresentation of gene ontology categories in biological networks Bioinformatics 2005 21 3448 3449 10.1093/bioinformatics/bti551 15972284

[B27] Li R Li Y Kristiansen K Wang J SOAP: Short oligonucleotide alignment program Bioinformatics 2008 24 713 714 10.1093/bioinformatics/btn025 18227114

[B28] Dewey CN Li B Ruotti V Stewart RM Thomson JA RNA-Seq gene expression estimation with read mapping uncertainty Bioinformatics 2010 26 493 500 10.1093/bioinformatics/btp692 20022975 PMC2820677

[B29] Harris RS Improved pairwise alignment of genomic DNA. Ph. D. Thesis 2007 The Pennsylvania State University,

[B30] Wang XW Wang LK Feng ZX Wang X Zhang XG DEGseq: an R package for identifying differentially expressed genes from RNA-seq data Bioinformatics 2010 26 136 138 10.1093/bioinformatics/btp612 19855105

[B31] Bateman A Birney E Durbin R Eddy SR Howe KL Sonnhammer ELL The Pfam protein families database Nucleic Acids Res 2000 28 263 266 10.1093/nar/28.1.263 10592242 PMC102420

[B32] Tatusov RL Koonin EV Lipman DJ A genomic perspective on protein families Science 1997 278 631 637 10.1126/science.278.5338.631 9381173

[B33] Cameron M Williams HE Cannane A Improved gapped alignment in BLAST Ieee Acm T Comput Bi 2004 1 116 129 10.1109/TCBB.2004.32 17048387

[B34] Munoz-Bertomeu J Cascales-Minana B Alaiz M Segura J Ros R A critical role of plastidial glycolytic glyceraldehyde-3-phosphate dehydrogenase in the control of plant metabolism and development Plant Signal Behav 2010 5 1 67 69 10.4161/psb.5.1.10200 20592814 PMC2835963

[B35] Munoz-Bertomeu J Cascales-Minana B Mulet JM Baroja-Fernandez E Pozueta-Romero J Kuhn JM Segura J Ros R Plastidial glyceraldehyde-3-phosphate dehydrogenase deficiency leads to altered root development and affects the sugar and amino acid balance in Arabidopsis Plant Physiol 2009 151 541 558 10.1104/pp.109.143701 19675149 PMC2754643

[B36] Lee Y Choi H Jin JY Choi S Hwang JU Kim YY Suh MC An ABCG/WBC-type ABC transporter is essential for transport of sporopollenin precursors for exine formation in developing pollen Plant J 2011 65 181 193 10.1111/j.1365-313X.2010.04412.x 21223384

[B37] Jiang LW Wang H Tse YC Law AHY Sun SSM Sun YB Xu ZF Hillmer S Robinson DG Vacuolar sorting receptors (VSRs) and secretory carrier membrane proteins (SCAMPs) are essential for pollen tube growth Plant J 2010 61 826 838 10.1111/j.1365-313X.2009.04111.x 20030753

[B38] Mellema S Eichenberger W Rawyler A Suter M Tadege M Kuhlemeier C The ethanolic fermentation pathway supports respiration and lipid biosynthesis in tobacco pollen Plant J 2002 30 329 336 10.1046/j.1365-313X.2002.01293.x 12000680

[B39] Rodriguez-Concepcion M Fores O Martinez-Garcia JF Gonzalez V Phillips MA Ferrer A Boronat A Distinct light-mediated pathways regulate the biosynthesis and exchange of isoprenoid precursors during Arabidopsis seedling development Plant Cell 2004 16 144 156 10.1105/tpc.016204 14660801 PMC301401

[B40] Ha SH Kim JB Hwang YS Lee SW Molecular characterization of three 3-hydroxy-3-methylglutaryl-CoA reductase genes including pathogen-induced Hmg2 from pepper (Capsicum annuum) Bba-Gene Struct Expr 2003 1625 253 260 10.1016/S0167-4781(02)00624-3 12591612

[B41] Chappell J Vonlanken C Vogeli U Elicitor-Inducible 3-Hydroxy-3-Methylglutaryl Coenzyme-a Reductase-Activity Is Required for Sesquiterpene Accumulation in Tobacco Cell-Suspension Cultures Plant Physiol 1991 97 693 698 10.1104/pp.97.2.693 16668454 PMC1081062

[B42] Arimura G Huber DP Bohlmann J Forest tent caterpillars (Malacosoma disstria) induce local and systemic diurnal emissions of terpenoid volatiles in hybrid poplar (Populus trichocarpa x deltoides): cDNA cloning, functional characterization, and patterns of gene expression of (−)-germacrene D synthase, PtdTPS1 Plant J 2004 37 603 616 10.1111/j.1365-313X.2003.01987.x 14756770

[B43] Zhuang X Klingeman WE Hu J Chen F Emission of volatile chemicals from flowering dogwood (cornus Florida L.) flowers J Agric Food Chem 2008 56 9570 9574 10.1021/jf801651v 18811168

[B44] Aharoni A Galili G Metabolic engineering of the plant primary-secondary metabolism interface Curr Opin Biotechnol 2011 22 239 244 10.1016/j.copbio.2010.11.004 21144730

[B45] Givan CV Evolving concepts in plant glycolysis: two centuries of progress Biol Rev 1999 74 277 309 10.1017/S0006323199005344

[B46] Hausler RE Prabhakar V Lottgert T Geimer S Dormann P Kruger S Vijayakumar V Schreiber L Gobel C Feussner K Phosphoenolpyruvate Provision to Plastids Is Essential for Gametophyte and Sporophyte Development in Arabidopsis thaliana Plant Cell 2010 22 2594 2617 10.1105/tpc.109.073171 20798327 PMC2947176

[B47] Vanderstraeten D Rodriguespousada RA Goodman HM Vanmontagu M Plant Enolase - Gene Structure, Expression, and Evolution Plant Cell 1991 3 719 735 1841726 10.1105/tpc.3.7.719 PMC160039

[B48] Angelin B Einarsson K Liljeqvist L Nilsell K Heller RA 3-Hydroxy-3-Methylglutaryl Coenzyme-a Reductase in Human-Liver Microsomes - Active and Inactive Forms and Cross-Reactivity with Antibody against Rat-Liver Enzyme J Lipid Res 1984 25 1159 1166 6084040

[B49] Morin RJ Noble NA Srikantaiah MV Modulation of 3-Hydroxy-3-Methyl Glutaryl Coa Reductase by 2,3-Diphosphoglyceric Acid Experientia 1984 40 953 955 10.1007/BF01946455 6468621

[B50] Ibsen KH, TP, Basabe J Properties of Rat Pyruvate Kinase Isozymes 1975 Academic Press, Inc, New York

[B51] Rohde A Morreel K Ralph J Goeminne G Hostyn V De Rycke R Kushnir S Van Doorsselaere J Joseleau JP Vuylsteke M Molecular phenotyping of the pal1 and pal2 mutants of Arabidopsis thaliana reveals far-reaching consequences on phenylpropanoid, amino acid, and carbohydrate metabolism Plant Cell 2004 16 2749 2771 10.1105/tpc.104.023705 15377757 PMC520969

[B52] Matt P Krapp A Haake V Mock HP Stitt M Decreased Rubisco activity leads to dramatic changes of nitrate metabolism, amino acid metabolism and the levels of phenylpropanoids and nicotine in tobacco antisense RBCS transformants Plant J 2002 30 663 677 10.1046/j.1365-313X.2002.01323.x 12061898

[B53] Moriizumi S Gourdon L Lefrancois-Martinez AM Kahn A Raymondjean M Effect of different basic helix-loop-helix leucine zipper factors on the glucose response unit of the L-type pyruvate kinase gene Gene Expr 1998 7 103 113 9699482 PMC6190201

[B54] Ma L Sham YY Walters KJ Towle HC A critical role for the loop region of the basic helix-loop-helix/leucine zipper protein Mlx in DNA binding and glucose-regulated transcription Nucleic Acids Res 2007 35 35 44 10.1093/nar/gkl730 17148476 PMC1761440

[B55] Li MV Chang B Imamura M Poungvarin N Chan L Glucose-dependent transcriptional regulation by an evolutionarily conserved glucose-sensing module Diabetes 2006 55 1179 1189 10.2337/db05-0822 16644671

[B56] Maffi D Iriti M Pigni M Vannini C Faoro F Uromyces appendiculatus infection in BTH-treated bean plants: ultrastructural details of a lost fight Mycopathologia 2011 171 209 221 10.1007/s11046-010-9350-1 20652832

[B57] Fukasawa-Akada T Kung SD Watson JC Phenylalanine ammonia-lyase gene structure, expression, and evolution in Nicotiana Plant Mol Biol 1996 30 711 722 10.1007/BF00019006 8624404

[B58] Nishihara M Nakatsuka T Yamamura S Flavonoid components and flower color change in transgenic tobacco plants by suppression of chalcone isomerase gene FEBS Lett 2005 579 6074 6078 10.1016/j.febslet.2005.09.073 16226261

[B59] Dobritsa AA Lei Z Nishikawa S Urbanczyk-Wochniak E Huhman DV Preuss D Sumner LW LAP5 and LAP6 encode anther-specific proteins with similarity to chalcone synthase essential for pollen exine development in Arabidopsis Plant Physiol 2010 153 937 955 10.1104/pp.110.157446 20442277 PMC2899912

[B60] Douglas CJ Souza CD Kim SS Koch S Kienow L Schneider K McKim SM Haughn G Kombrink E A Novel Fatty Acyl-CoA Synthetase Is Required for Pollen Development and Sporopollenin Biosynthesis in Arabidopsis Plant Cell 2009 21 507 525 10.1105/tpc.108.062513 19218397 PMC2660628

[B61] Komatsuda T Sakuma S Pourkheirandish M Matsumoto T Koba T Duplication of a well-conserved homeodomain-leucine zipper transcription factor gene in barley generates a copy with more specific functions Funct Integr Genomic 2010 10 123 133 10.1007/s10142-009-0134-y PMC2834773 19707806

