# Supplementary material for: Genetic variation and metabolic pathway intricacy govern the active compound content and quality of the Chinese medicinal plantLonicera japonicathunb
Source: BMC Genomics. 2012 May 20;13:195. doi: 10.1186/1471-2164-13-195 (PMC3443457; doi:10.1186/1471-2164-13-195)
Supplement: Additional file 1 — Figure S1. Comparing volatile compound of FLJ and rFLJ using Gas chromatography-mass spectrometry. A, Bud, B, flower1, C, flower2. Black line represent FLJ and Red line represent rFLJ. FLJ, Lonicera japonica Thumb; rFLJ, Lonicera japonica Thunb. var. chinensis (Wats.). Figure S2. HPLC analysis of active compounds in FLJ and rFLJ. A, Mix of standard compounds. Chlorogenic acid (RT, 13.20; MW, 354.31); Caffeic acid (RT, 17.39; MW, 180.15); ferulic acid (RT, 24.24; MW, 194.18); Rutin (RT, 24.24; MW, 610.52); Luteoloside(RT, 25.27; MW,448.4); Hyperoside (RT, 25.35; MW, 464.37); Quercitrin (RT, 28.65; MW, 448.38); Quercetin (RT, 38.04; MW, 302.24). B, buds of rFLJ; C, buds of FLJ. FLJ, Lonicera japonica Thumb; rFLJ, Lonicera japonica Thunb. var. chinensis (Wats.). Figure S3. BlastX analysis result of contigs in FLJ and rFLJ with all non-redundant (NR) database in Genebank. Six species,Vitis vinifera, Ricinus communis, Populus trichocarpa, Glycine max, Arabidopsis lyrata, Nicotiana tabacum has highest identity with FLJ and rFLJ bud. E-value cut-off was lower than 1e-5. FLJ, Lonicera japonica Thumb; rFLJ, Lonicera japonica Thunb. var. chinensis (Wats.). Figure S4. Pathways of major chemical compounds inLonicera japonicaThumb (FLJ). All of contigs from three FLJ libraries were annotated with KEGG database. The six pathways, phenylalanine metabolism, terpenoid backbone, fatty acid biosynthesis, citric acid cycle, glycolysis and sucrose metabolism were analysis. Green rectangles repress enzymes finding in FLJ transcriptome. Figure S5. Analysis of gene differential express using MA-plot-based method. M is the Y -axis and represents the intensity ratio, and A is the X-axis and represents the average intensity for each transcript. The red points are the genes identity as differentially expressed. FLJ, Lonicera japonica Thumb; rFLJ, Lonicera japonica Thunb. var. chinensis (Wats.). Figure S6 Pathway assignment based on KEGG analysis of differential express genes between buds and o [file 1471-2164-13-195-S1.ppt]

## Slide 1
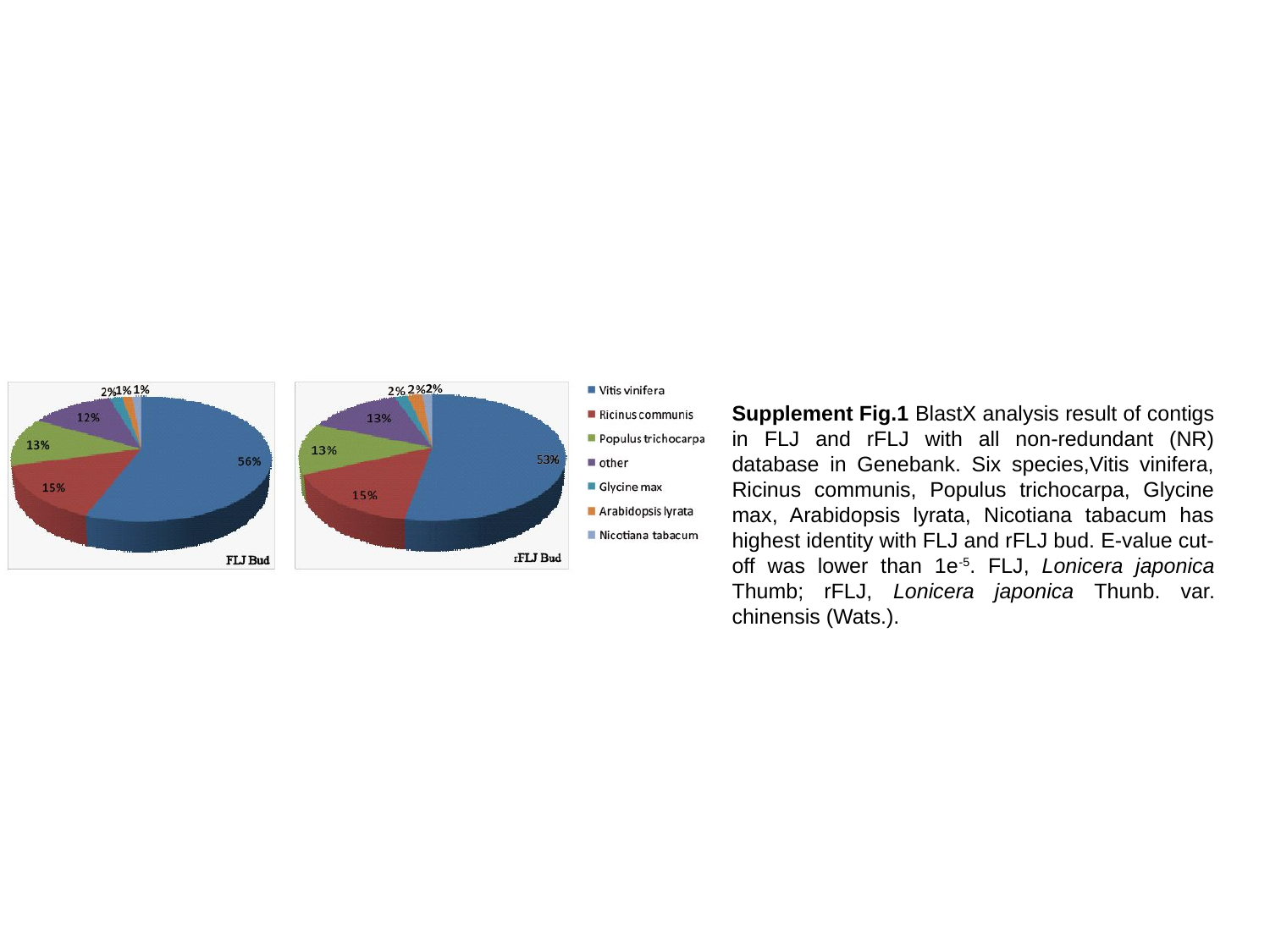

Supplement Fig.1 BlastX analysis result of contigs in FLJ and rFLJ with all non-redundant (NR) database in Genebank. Six species,Vitis vinifera, Ricinus communis, Populus trichocarpa, Glycine max, Arabidopsis lyrata, Nicotiana tabacum has highest identity with FLJ and rFLJ bud. E-value cut-off was lower than 1e-5. FLJ, Lonicera japonica Thumb; rFLJ, Lonicera japonica Thunb. var. chinensis (Wats.).

## Slide 2
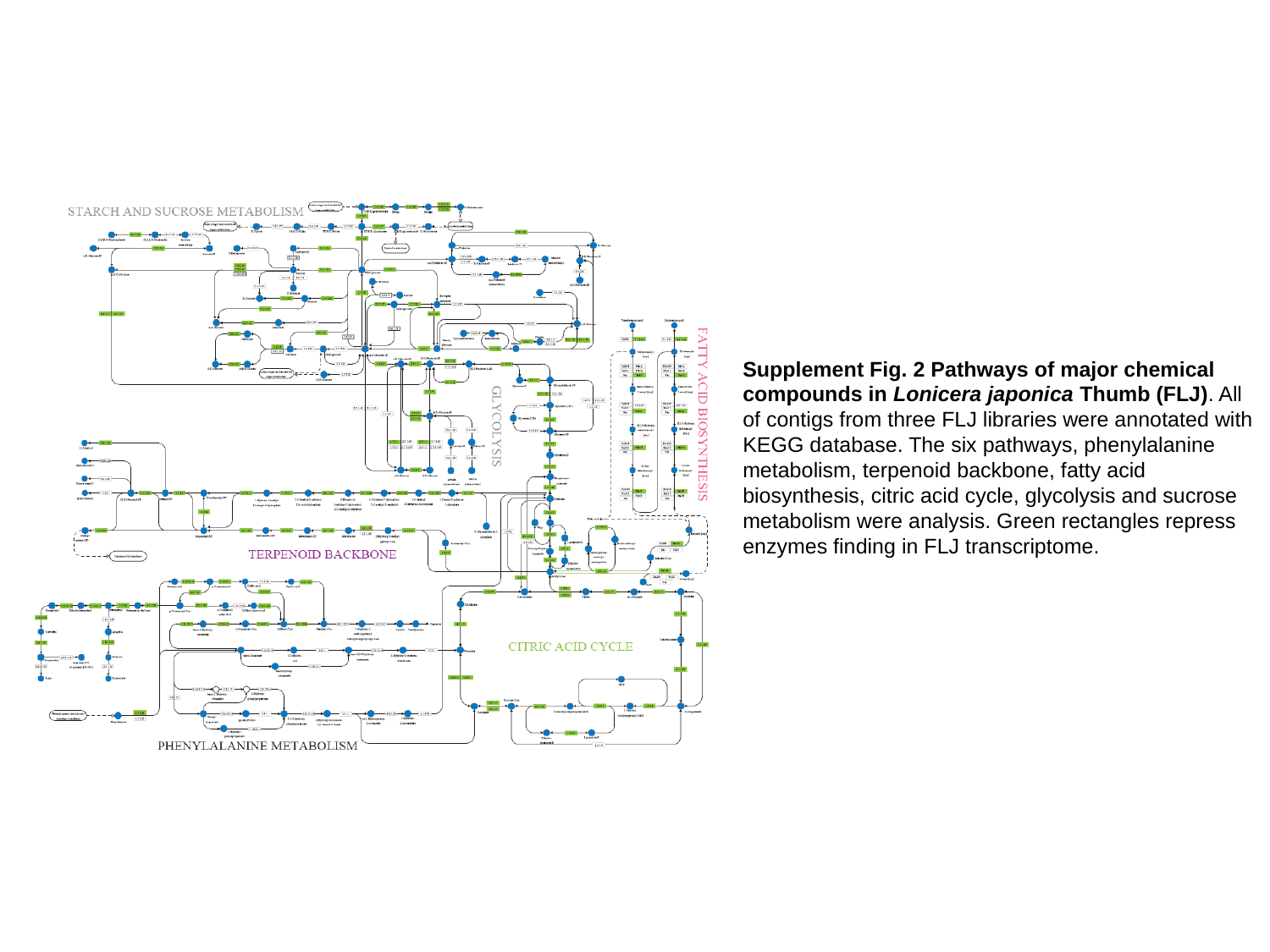

Supplement Fig. 2 Pathways of major chemical compounds in Lonicera japonica Thumb (FLJ). All of contigs from three FLJ libraries were annotated with KEGG database. The six pathways, phenylalanine metabolism, terpenoid backbone, fatty acid biosynthesis, citric acid cycle, glycolysis and sucrose metabolism were analysis. Green rectangles repress enzymes finding in FLJ transcriptome.

## Slide 3
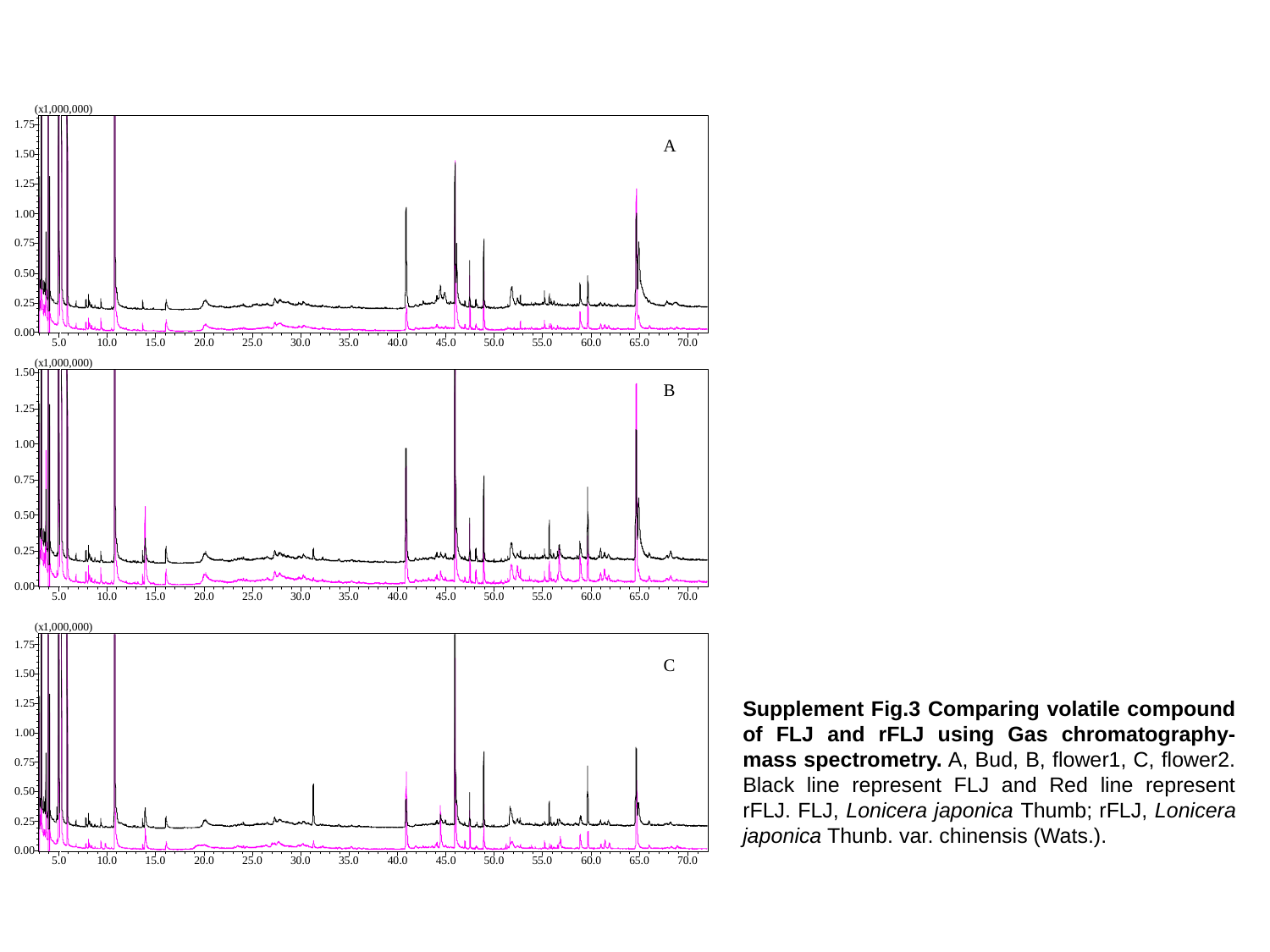

A
B
C
Supplement Fig.3 Comparing volatile compound of FLJ and rFLJ using Gas chromatography-mass spectrometry. A, Bud, B, flower1, C, flower2. Black line represent FLJ and Red line represent rFLJ. FLJ, Lonicera japonica Thumb; rFLJ, Lonicera japonica Thunb. var. chinensis (Wats.).

## Slide 4
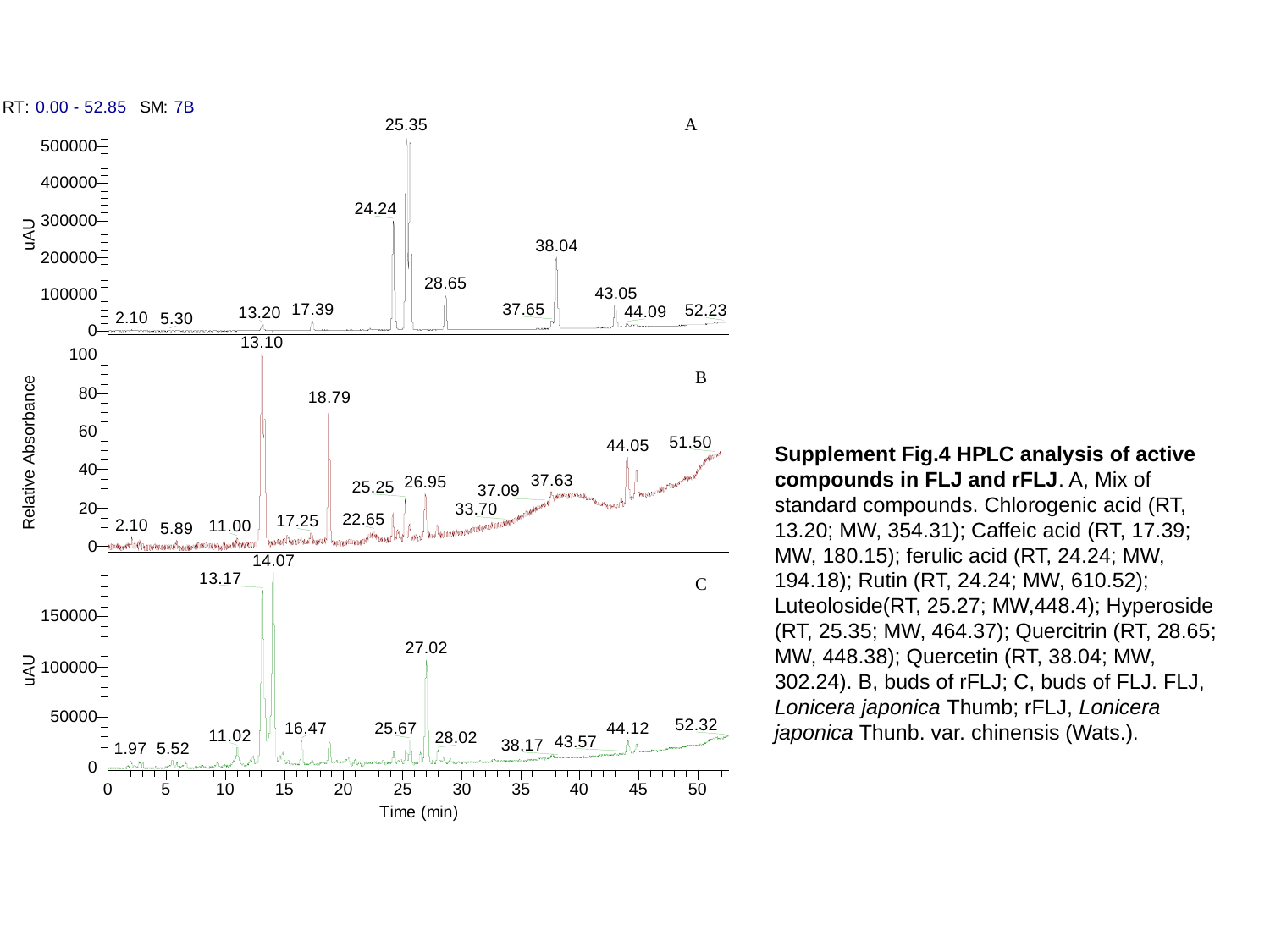

A
B
Supplement Fig.4 HPLC analysis of active compounds in FLJ and rFLJ. A, Mix of standard compounds. Chlorogenic acid (RT, 13.20; MW, 354.31); Caffeic acid (RT, 17.39; MW, 180.15); ferulic acid (RT, 24.24; MW, 194.18); Rutin (RT, 24.24; MW, 610.52); Luteoloside(RT, 25.27; MW,448.4); Hyperoside (RT, 25.35; MW, 464.37); Quercitrin (RT, 28.65; MW, 448.38); Quercetin (RT, 38.04; MW, 302.24). B, buds of rFLJ; C, buds of FLJ. FLJ, Lonicera japonica Thumb; rFLJ, Lonicera japonica Thunb. var. chinensis (Wats.).
C

## Slide 5
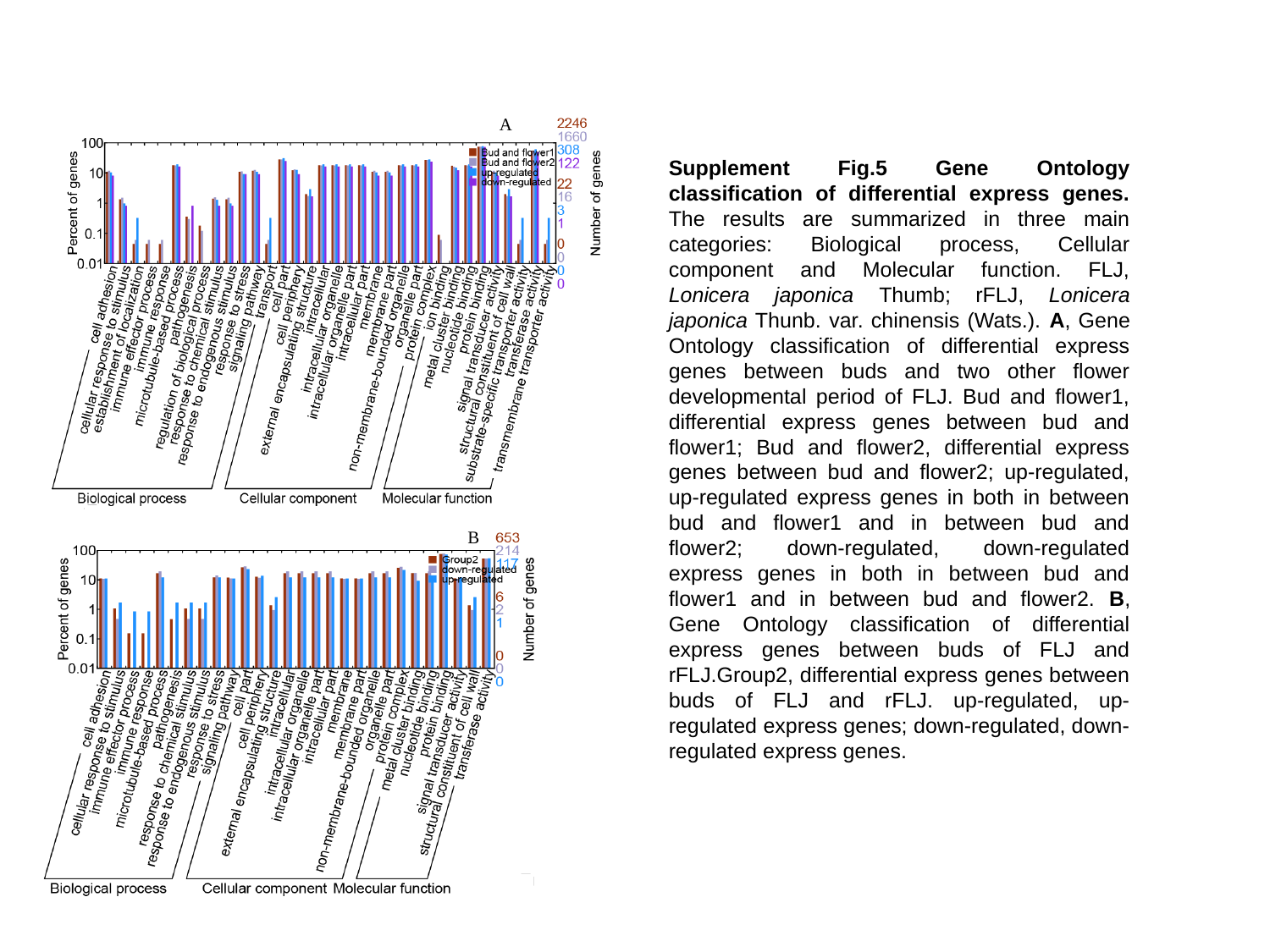

A
Supplement Fig.5 Gene Ontology classification of differential express genes. The results are summarized in three main categories: Biological process, Cellular component and Molecular function. FLJ, Lonicera japonica Thumb; rFLJ, Lonicera japonica Thunb. var. chinensis (Wats.). A, Gene Ontology classification of differential express genes between buds and two other flower developmental period of FLJ. Bud and flower1, differential express genes between bud and flower1; Bud and flower2, differential express genes between bud and flower2; up-regulated, up-regulated express genes in both in between bud and flower1 and in between bud and flower2; down-regulated, down-regulated express genes in both in between bud and flower1 and in between bud and flower2. B, Gene Ontology classification of differential express genes between buds of FLJ and rFLJ.Group2, differential express genes between buds of FLJ and rFLJ. up-regulated, up-regulated express genes; down-regulated, down-regulated express genes.
B

## Slide 6
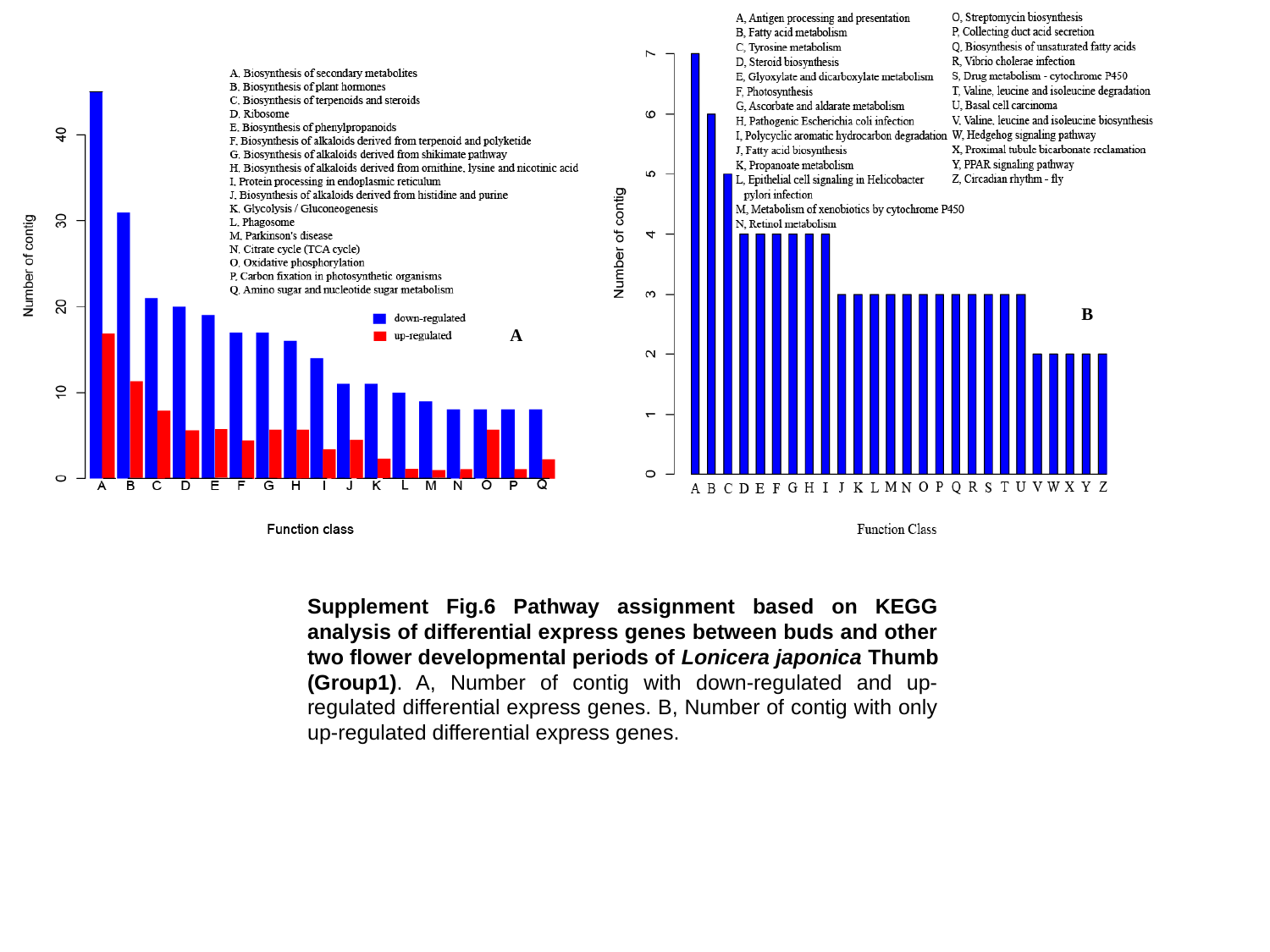

B
A
Supplement Fig.6 Pathway assignment based on KEGG analysis of differential express genes between buds and other two flower developmental periods of Lonicera japonica Thumb (Group1). A, Number of contig with down-regulated and up-regulated differential express genes. B, Number of contig with only up-regulated differential express genes.

## Slide 7
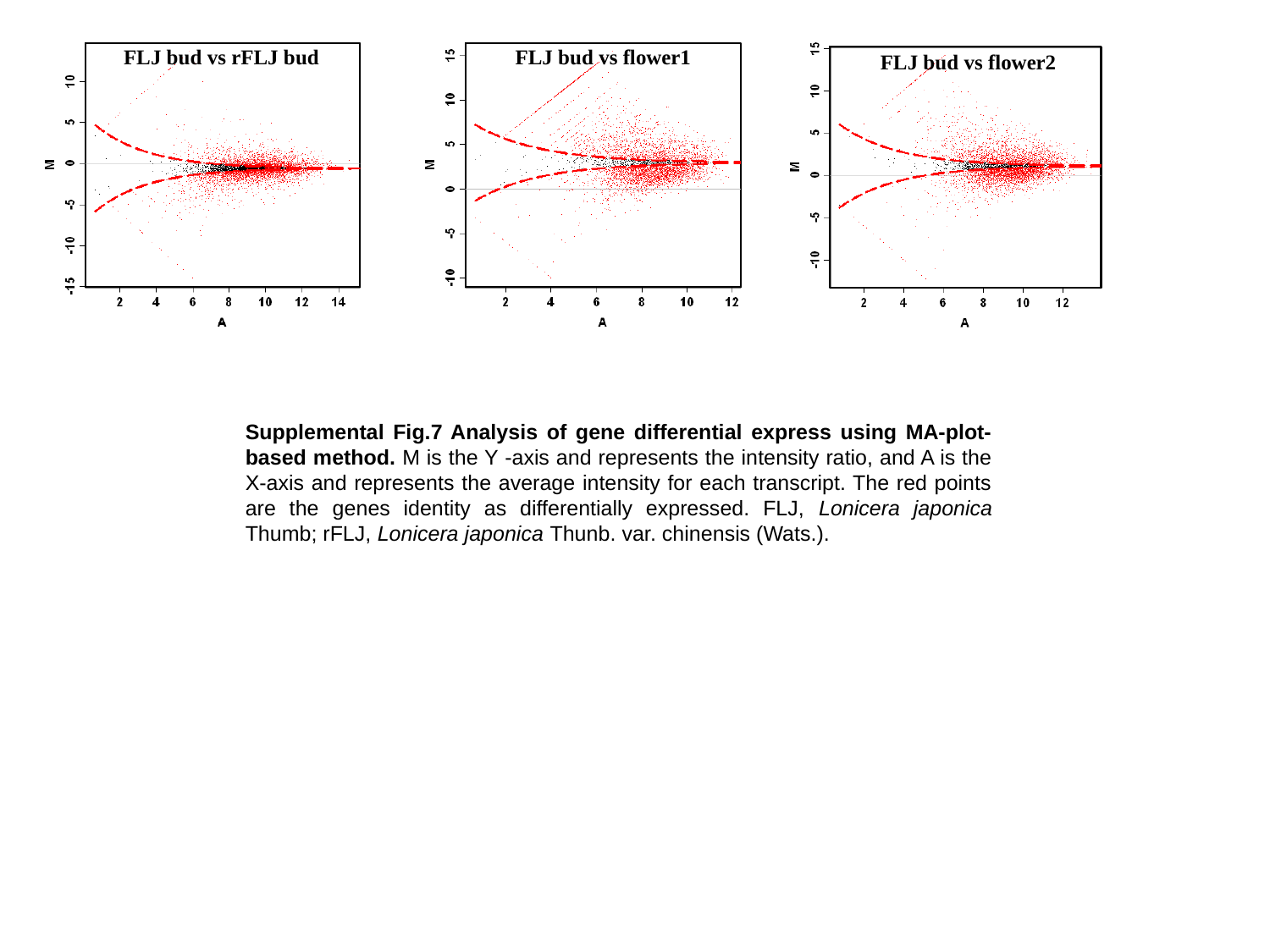

FLJ bud vs rFLJ bud
FLJ bud vs flower1
FLJ bud vs flower2
Supplemental Fig.7 Analysis of gene differential express using MA-plot-based method. M is the Y -axis and represents the intensity ratio, and A is the X-axis and represents the average intensity for each transcript. The red points are the genes identity as differentially expressed. FLJ, Lonicera japonica Thumb; rFLJ, Lonicera japonica Thunb. var. chinensis (Wats.).

## Slide 8
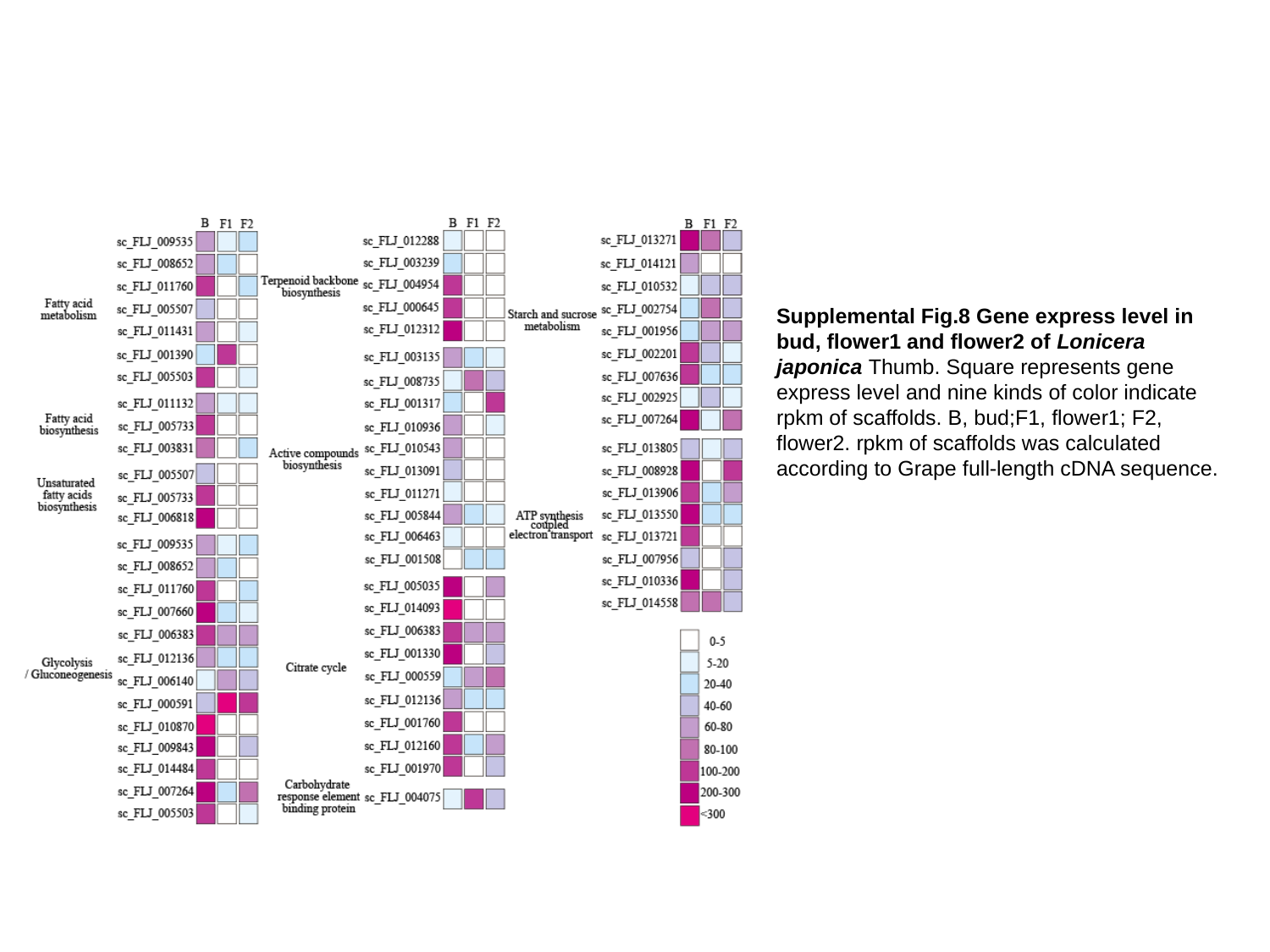

Supplemental Fig.8 Gene express level in bud, flower1 and flower2 of Lonicera japonica Thumb. Square represents gene express level and nine kinds of color indicate rpkm of scaffolds. B, bud;F1, flower1; F2, flower2. rpkm of scaffolds was calculated according to Grape full-length cDNA sequence.

## Slide 9
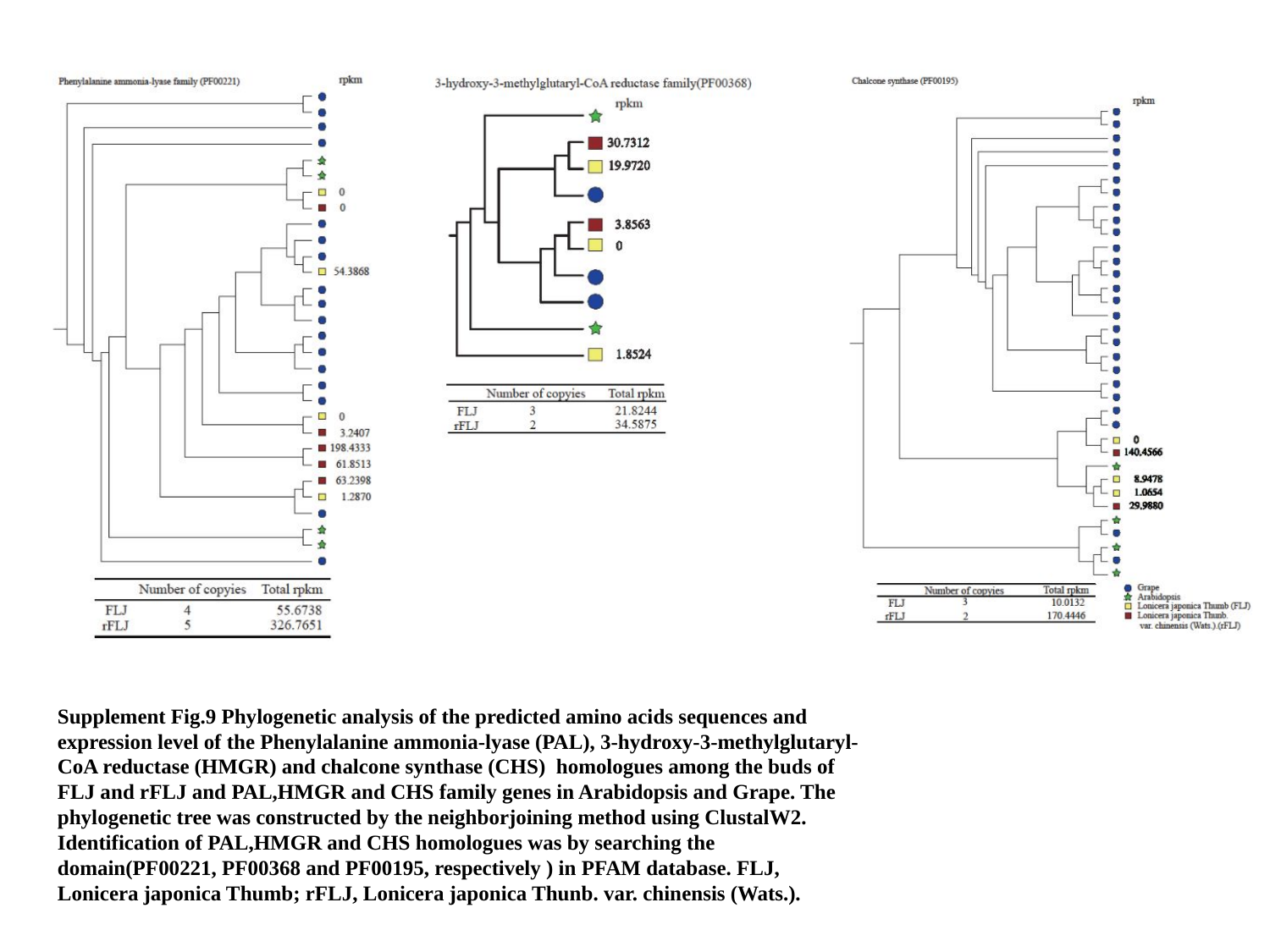

Supplement Fig.9 Phylogenetic analysis of the predicted amino acids sequences and expression level of the Phenylalanine ammonia-lyase (PAL), 3-hydroxy-3-methylglutaryl-CoA reductase (HMGR) and chalcone synthase (CHS) homologues among the buds of FLJ and rFLJ and PAL,HMGR and CHS family genes in Arabidopsis and Grape. The phylogenetic tree was constructed by the neighborjoining method using ClustalW2. Identification of PAL,HMGR and CHS homologues was by searching the domain(PF00221, PF00368 and PF00195, respectively ) in PFAM database. FLJ, Lonicera japonica Thumb; rFLJ, Lonicera japonica Thunb. var. chinensis (Wats.).
