# Supplementary material for: Genetic variation and metabolic pathway intricacy govern the active compound content and quality of the Chinese medicinal plantLonicera japonicathunb
Source: BMC Genomics. 2012 May 20;13:195. doi: 10.1186/1471-2164-13-195 (PMC3443457; doi:10.1186/1471-2164-13-195)
Supplement: Additional file 2 — Table S1. The Elution Conditions of HPLC Analysis. Note: T, Retention time; A, mobile phase deionized water- formic acid (99:1, v/v); B, mobile phase methanol. Table S2. Formula of Active Compound Content. Note: Calibration plots of eight standards were constructed on the basis of peak areas (y) using seven different concentration solutions (x). All plots were linear in the examined ranges, and linear ranges had been shown as the concentration of the standard compounds (μg mL-1). The r referred to the correlation coefficient of the equation. The standard compounds Chlorogenic acid (110753), Caffeic acid(110885), ferulic acid(110773), Rutin(100080), Luteoloside(111720), Hyperoside(111521), Quercitrin(111538) and Quercetin (100081) were purchased from National institutes for food and drug control, China. Table S3. Number of Contigs in KEGG Pathways. Note: FLJ, Lonicera japonica Thumb; rFLJ, Lonicera japonica Thunb. var. chinensis (Wats.); B, bud; F1,flower1; F2, flower2. Table S4. Predicted Phosphorylated Sites in 34 Protein Sequence from Differential Express Gene. Note: Predicted Phosphorylated Sites using software online (http://kinasephos2.mbc.nctu.edu.tw/) and protein sequence was perdicted by ORF finder (http://www.ncbi.nlm.nih.gov/gorf/gorf.html). Table S5. qRT-PCR and RNA-seq Analysis of Gene Express between Buds ofLonicera japonicaThunb. var. chinensis (Wats.) andLonicera japonicaThumb. Note: RR/YR, the ratio of transcripted level in buds of Lonicera japonica Thunb. var. chinensis (Wats.) and Lonicera japonica Thumb. Table S6. Orthologs Identity of Differential Express Genes Sequence between FLJ and rFLJ. Note: FLJ, Lonicera japonica Thumb; rFLJ, Lonicera japonica Thunb. var. chinensis (Wats.) Table S7 PAL, CHS, HMGR and PK Gene Families in Arabidopsis and Grape. Table S8. Putative Enzyme Pool to Control the Active Compounds in Buds ofLonicera japonicaThumb. [file 1471-2164-13-195-S2.doc]

**Supplement Table 1 The Elution Conditions of HPLC Analysis**

| **T (min)** | **A (%)** | **B (%)** |
| --- | --- | --- |
| 0 | 95 | 5 |
| 5 | 95 | 5 |
| 15 | 90 | 10 |
| 25 | 90 | 10 |
| 40 | 75 | 25 |
| 44 | 65 | 35 |
| 45 | 50 | 50 |
| 50 | 45 | 55 |
| 55 | 0 | 100 |
| 60 | 0 | 100 |

Note: T, Retention time; A, mobile phase deionized water- formic acid ( 99:1, v/v); B, mobile phase methanol.

**Supplement table 2 Formula of Active Compound Content**

| **Compounds** | **Formula** | **Linearity correlation (R)** |
| --- | --- | --- |
| Chlorogenic acid | Y=200.3X-10.7 | 0.9997 |
| Caffeic acid | Y=282.1X-12.8 | 0.9996 |
| Ferulic acid | Y=210.8X+1.2 | 0.9998 |
| Rutin | Y=174.5X-6.3 | 0.9995 |
| Hyperoside | Y=180.6X-5.4 | 0.9994 |
| Luteoloside | Y=178.4X-4.8 | 0.9995 |
| Quercitrin | Y=190.2X-6.1 | 0.9996 |
| Quercetin | Y=187.2X-7.1 | 0.9997 |

Note: Calibration plots of eight standards were constructed on the basis of peak areas (y) using seven different concentration solutions (x). All plots were linear in the examined ranges, and linear ranges had been shown as the concentration of the standard compounds (μg mL-1). The r referred to the correlation coefficient of the equation. The standard compounds Chlorogenic acid (110753), Caffeic acid(110885), ferulic acid(110773), Rutin(100080), Luteoloside(111720), Hyperoside(111521), Quercitrin(111538) and Quercetin (100081) were purchased from National institutes for food and drug control, China.

**Supplement table 3. Number of Contigs in KEGG Pathways**

| **Pathways** | | **FLJ Bud** | **FLJ F1** | **FLJ F2** | **rFLJ Bud** | **rFLJ F2** |
| --- | --- | --- | --- | --- | --- | --- |
| **map01100** | Metabolic pathways | 674 | 626 | 656 | 652 | 667 |
| **map01110** | Biosynthesis of secondary metabolites | 273 | 245 | 265 | 271 | 267 |
| **map01070** | Biosynthesis of plant hormones | 115 | 101 | 109 | 115 | 114 |
| **map03010** | Ribosome | 109 | 115 | 110 | 108 | 110 |
| **map03040** | Spliceosome | 94 | 92 | 94 | 94 | 83 |
| **map00190** | Oxidative phosphorylation | 85 | 81 | 82 | 83 | 91 |
| **map01062** | Biosynthesis of terpenoids and steroids | 74 | 64 | 70 | 74 | 76 |
| **map00230** | Purine metabolism | 71 | 66 | 72 | 63 | 66 |
| **map05016** | Huntington's disease | 68 | 62 | 64 | 66 | 68 |
| **map01061** | Biosynthesis of phenylpropanoids | 67 | 63 | 62 | 67 | 62 |
| **map01063** | Biosynthesis of alkaloids derived from shikimate pathway | 60 | 57 | 57 | 58 | 58 |
| **map04141** | Protein processing in endoplasmic reticulum | 59 | 59 | 65 | 57 | 74 |
| **map01064** | Biosynthesis of alkaloids derived from ornithine, lysine and nicotinic acid | 58 | 56 | 59 | 58 | 58 |
| **map00240** | Pyrimidine metabolism | 57 | 53 | 55 | 47 | 51 |
| **map01065** | Biosynthesis of alkaloids derived from histidine and purine | 56 | 55 | 56 | 56 | 55 |
| **map05012** | Parkinson's disease | 55 | 50 | 53 | 53 | 57 |
| **map04120** | Ubiquitin mediated proteolysis | 54 | 51 | 52 | 48 | 48 |
| **map01066** | Biosynthesis of alkaloids derived from terpenoid and polyketide | 53 | 47 | 49 | 53 | 52 |
| **map05010** | Alzheimer's disease | 53 | 49 | 50 | 52 | 55 |
| **map03018** | RNA degradation | 44 | 39 | 41 | 38 | 37 |

**Note:** FLJ, *Lonicera japonica* Thumb; rFLJ, *Lonicera japonica* Thunb. var. chinensis (Wats.); B, bud; F1,flower1; F2, flower2;

**Supplement table 4. Predicted Phosphorylated Sites in 34 Protein Sequence from Differential Express Gene**

| **Protein Name** | **Predicted Phosphorylated Sites** | | |
| --- | --- | --- | --- |
|  | **Serine** | **Threonine** | **Tyrosine** |
| sc_FLJ_014774 | 4 | 5 | 1 |
| sc_FLJ_011370 | 6 | 2 | 1 |
| sc_FLJ_012136 | 13 | 2 | 1 |
| sc_FLJ_006268 | 13 | 6 | 2 |
| sc_FLJ_005938 | 1 | 2 | 1 |
| sc_FLJ_000014 | 2 | 5 | 3 |
| sc_FLJ_000591 | 2 | 1 | 1 |
| sc_FLJ_007617 | 5 | 3 | 1 |
| sc_FLJ_012087 | 5 | 3 | 4 |
| sc_FLJ_010733 | 4 | 1 | 1 |
| sc_FLJ_013089 | 2 | 1 | 3 |
| sc_FLJ_003586 | 4 | 3 | 2 |
| sc_FLJ_002927 | 3 | 3 | 3 |
| sc_FLJ_008735 | 0 | 0 | 1 |
| sc_FLJ_009590 | 3 | 1 | 0 |
| sc_FLJ_011271 | 2 | 2 | 2 |
| [sc_FLJ_003239](http://kinasephos.mbc.nctu.edu.tw/predict.php#sc_FLJ_003239) | 5 | 0 | 3 |
| [sc_FLJ_003831](http://kinasephos.mbc.nctu.edu.tw/predict.php#sc_FLJ_003831) | 8 | 3 | 0 |
| [sc_FLJ_012288](http://kinasephos.mbc.nctu.edu.tw/predict.php#sc_FLJ_012288) | 6 | 2 | 1 |
| sc_FLJ_005423 | 4 | 2 | 3 |
| sc_FLJ_013271 | 2 | 5 | 2 |
| sc_FLJ_008054 | 4 | 2 | 3 |
| sc_FLJ_013965 | 1 | 1 | 5 |
| sc_FLJ_005429 | 11 | 3 | 8 |
| sc_FLJ_007264 | 7 | 3 | 5 |
| sc_FLJ_009499 | 3 | 3 | 0 |
| sc_FLJ_007450 | 3 | 0 | 1 |
| sc_FLJ_001317 | 5 | 2 | 1 |
| sc_FLJ_010476 | 6 | 4 | 5 |
| sc_FLJ_014093 | 4 | 2 | 3 |
| sc_FLJ_006617 | 5 | 2 | 0 |
| sc_FLJ_011483 | 3 | 2 | 0 |
| sc_FLJ_000185 | 3 | 0 | 2 |
| sc_FLJ_012312 | 2 | 2 | 2 |

Note: Predicted Phosphorylated Sites using software online (http://kinasephos2.mbc.nctu.edu.tw/) and protein sequence was perdicted by ORF finder (http://www.ncbi.nlm.nih.gov/gorf/gorf.html).

**Supplement Table 5. qRT-PCR and RNA-seq Analysis of Gene Express between Buds of *Lonicera japonica* Thunb. var. chinensis (Wats.) and *Lonicera japonica* Thumb..**

| Scafforld | Primers | | RR/YR | |
| --- | --- | --- | --- | --- |
|  | L (5’-3’) | R (5’-3’) | qRT-PCR | RNA-seq |
| sc_FLJ_013089 | TATCCTTCAAGGCAGCGACTA | CCAGTAATAAGCACAGTTCCGA | 1.6 | 2.8 |
| sc_FLJ_008735 | TAGTTGTCCTTGTCAGCGTCC | CCAGCGTGTATCCAAGAGAAC | 5.6 | 3.0 |
| sc_FLJ_001317 | TTCCTGTGGAACCTGAATGTC | CTAATGGTGACCTCCGATCAA | 2.7 | 3.8 |
| sc_FLJ_008421 | CAGGTCCAAGGCATGTTAGAG | TGCCAAGATATCAGACTTCGG | 1.6 | 3.5 |
| sc_FLJ_004075 | GAATCTTGGAACAGCACGAAG | GGTTGTCCACTCCTACCATTG | 1.7 | 1.1 |
| sc_FLJ_012312 | CATTGTGAAGAACATGGCTGA | TACATAGCATCTTGCCGCTCT | 5.3 | 2.1 |
| sc_FLJ_002416 | GTACTCGAAGCAGAGCAGCAT | GATTCATCATTGGCTCCTGAA | 0.3 | 0.3 |
| sc_FLJ_013965 | GCGTACTACTCCGCAACCATA | CCAATCTTGCCAATAACCTCA | 0.1 | 0.5 |
| sc_FLJ_003239 | TGGAGCTGCCTATTGTACTGG | ACGCGTTATTGGTTGAGAATG | 1.5 | 2.9 |
| sc_FLJ_004954 | GACGAACACGTGCTGTAGTCA | TAGCTTCAGACCACCATAGCC | 2.9 | 3.1 |
| TubRL | TGGCCGCTACCTCACTGCCT | TCGTCCATGCCTTCCCCGGT | - | - |

Note: RR/YR, the ratio of transcripted level in buds of *Lonicera japonica* Thunb. var. chinensis (Wats.) and *Lonicera japonica* Thumb.

**Supplement table 6. Orthologs Identity of Differential Express Genes Sequence between FLJ and rFLJ.**

| Contig | | Scafford | Identity(%) | Enzyme |
| --- | --- | --- | --- | --- |
| FLJB192692 | rFLJB144680 | sc_FLJ_010942 | 97.4 | aldose 1-epimerase [EC:5.1.3.3] |
| FLJB186872 | rFLJB152487 | sc_FLJ_014093 | 97.7 | malate dehydrogenase [EC:1.1.1.37] |
| FLJB187861 | rFLJB147888 | sc_FLJ_014428 | 97.8 | omega-3 fatty acid desaturase (delta-15 desaturase) [EC:1.14.19.-] |
| FLJB194726 | rFLJB150804 | sc_FLJ_003831 | 98.0 | fatty acyl-ACP thioesterase B [EC:3.1.2.14 3.1.2.-] |
| FLJB192724 | rFLJB136278 | sc_FLJ_005423 | 98.0 | fructose-bisphosphate aldolase, class I [EC:4.1.2.13] |
| FLJB54966 | rFLJB154119 | sc_FLJ_001317 | 98.3 | 4-coumarate--CoA ligase [EC:6.2.1.12] |
| FLJB192366 | rFLJB146435 | sc_FLJ_008735 | 98.5 | caffeoyl-CoA O-methyltransferase [EC:2.1.1.104] |
| FLJB184991 | rFLJB148476 | sc_FLJ_007264 | 98.8 | phosphoglucomutase [EC:5.4.2.2] |
| FLJB184086 | rFLJB153556 | sc_FLJ_000720 | 98.8 | hexokinase [EC:2.7.1.1] |
| FLJB132964 | rFLJB144990 | sc_FLJ_003239 | 98.8 | 4-diphosphocytidyl-2-C-methyl-D-erythritol kinase [EC:2.7.1.148] |
| FLJB185230 | rFLJB152616 | sc_FLJ_009020 | 98.8 | trans-cinnamate 4-monooxygenase [EC:1.14.13.11] |
| FLJB10821 | rFLJB145029 | sc_FLJ_000185 | 99.1 | ATP citrate (pro-S)-lyase [EC:2.3.3.8] |
| FLJB182389 | rFLJB136661 | sc_FLJ_005015 | 99.1 | TTG1 |
| FLJB191890 | rFLJB152274 | sc_FLJ_001232 | 99.2 | phenylalanine ammonia-lyase [EC:4.3.1.24] |
| FLJB187369 | rFLJB152558 | sc_FLJ_000721 | 99.2 | hexokinase [EC:2.7.1.1] |
| FLJB185374 | rFLJB146338 | sc_FLJ_000591 | 99.2 | pyruvate kinase [EC:2.7.1.40] |
| FLJB194566 | rFLJB145788 | sc_FLJ_011795 | 99.3 | dihydrolipoamide dehydrogenase [EC:1.8.1.4] |
| FLJB184017 | rFLJB136694 | sc_FLJ_010476 | 99.3 | acetyl-CoA synthetase [EC:6.2.1.1] |
| FLJB38721 | rFLJB150937 | sc_FLJ_012312 | 99.3 | 4-hydroxy-3-methylbut-2-enyl diphosphate reductase [EC:1.17.1.2] |
| FLJB184290 | rFLJB153109 | sc_FLJ_012225 | 99.3 | 1-deoxy-D-xylulose-5-phosphate synthase [EC:2.2.1.7] |
| FLJB163426 | rFLJB26408 | sc_FLJ_006617 | 99.5 | beta-carotene hydroxylase [EC:1.14.13.-] |
| FLJB192057 | rFLJB25324 | sc_FLJ_000290 | 99.5 | UDPglucose 6-dehydrogenase [EC:1.1.1.22] |
| FLJB192051 | rFLJB136268 | sc_FLJ_002927 | 99.5 | fructose-bisphosphate aldolase, class I [EC:4.1.2.13] |
| FLJB139259 | rFLJB148037 | sc_FLJ_012136 | 99.5 | dihydrolipoamide dehydrogenase [EC:1.8.1.4] |
| FLJB190312 | rFLJB150202 | sc_FLJ_013091 | 99.6 | phenylalanine ammonia-lyase [EC:4.3.1.24] |
| FLJB185861 | rFLJB153983 | sc_FLJ_001109 | 99.6 | beta-glucosidase [EC:3.2.1.21] |
| FLJB192796 | rFLJB58398 | sc_FLJ_000291 | 99.6 | UDPglucose 6-dehydrogenase [EC:1.1.1.22] |
| FLJB93452 | rFLJB151486 | sc_FLJ_006268 | 99.7 | pyruvate kinase [EC:2.7.1.40] |
| FLJB18975 | rFLJB150600 | sc_FLJ_012145 | 99.7 | 1-deoxy-D-xylulose-5-phosphate synthase [EC:2.2.1.7] |
| FLJB184284 | rFLJB149659 | sc_FLJ_008054 | 99.7 | enolase [EC:4.2.1.11] |
| FLJB193084 | rFLJB146541 | sc_FLJ_004955 | 99.7 | isopentenyl-diphosphate delta-isomerase [EC:5.3.3.2] |
| FLJB190742 | rFLJB146405 | sc_FLJ_014774 | 99.7 | pyruvate kinase [EC:2.7.1.40] |
| FLJB182466 | rFLJB144642 | sc_FLJ_011483 | 99.7 | hexokinase [EC:2.7.1.1] |
| FLJB186848 | rFLJB149535 | sc_FLJ_010189 | 99.7 | pyruvate kinase [EC:2.7.1.40] |
| FLJB183911 | rFLJB146659 | sc_FLJ_000014 | 99.8 | beta-glucosidase [EC:3.2.1.21] |
| FLJB190939 | rFLJB151284 | sc_FLJ_013966 | 99.8 | farnesyl diphosphate synthase [EC:2.5.1.1 2.5.1.10] |
| FLJB190422 | rFLJB136003 | sc_FLJ_013089 | 99.8 | enoyl reductase [EC:1.3.1.-] |
| FLJB172027 | rFLJB135584 | sc_FLJ_007617 | 99.8 | omega-3 fatty acid desaturase (delta-15 desaturase) [EC:1.14.19.-] |
| FLJB186672 | rFLJB146108 | sc_FLJ_007265 | 99.8 | phosphoglucomutase [EC:5.4.2.2] |
| FLJB183876 | rFLJB101045 | sc_FLJ_005512 | 99.9 | fructose-bisphosphate aldolase, class I [EC:4.1.2.13] |
| FLJB184450 | rFLJB147181 | sc_FLJ_004954 | 99.9 | isopentenyl-diphosphate delta-isomerase [EC:5.3.3.2] |
| FLJB185235 | rFLJB150772 | sc_FLJ_010188 | 99.9 | pyruvate kinase [EC:2.7.1.40] |
| FLJB188972 | rFLJB147006 | sc_FLJ_013965 | 99.9 | farnesyl diphosphate synthase [EC:2.5.1.1 2.5.1.10] |
| FLJB194868 | rFLJB136765 | sc_FLJ_007266 | 99.9 | phosphoglucomutase [EC:5.4.2.2] |
| FLJB194703 | rFLJB145555 | sc_FLJ_003586 | 99.9 | hexokinase [EC:2.7.1.1] |
| FLJB186217 | rFLJB149466 | sc_FLJ_007450 | 99.9 | omega-3 fatty acid desaturase (delta-15 desaturase) [EC:1.14.19.-] |
| FLJB5229 | rFLJB8432 | sc_FLJ_013780 | 100.0 | 1-deoxy-D-xylulose-5-phosphate synthase [EC:2.2.1.7] |
| FLJB182684 | rFLJB145861 | sc_FLJ_000186 | 100.0 | ATP citrate (pro-S)-lyase [EC:2.3.3.8] |
| FLJB191828 | rFLJB150736 | sc_FLJ_000187 | 100.0 | ATP citrate (pro-S)-lyase [EC:2.3.3.8] |
| FLJB186518 | rFLJB153546 | sc_FLJ_014380 | 100.0 | caffeoyl-CoA O-methyltransferase [EC:2.1.1.104] |
| FLJB172918 | rFLJB135827 | sc_FLJ_009590 | 100.0 | naringenin 3-dioxygenase [EC:1.14.11.9] |
| FLJB184188 | rFLJB154084 | sc_FLJ_010733 | 100.0 | phenylalanine ammonia-lyase [EC:4.3.1.24] |
| FLJB186140 | rFLJB150726 | sc_FLJ_010734 | 100.0 | phenylalanine ammonia-lyase [EC:4.3.1.24] |
| FLJB112959 | rFLJB46493 | sc_FLJ_002513 | 100.0 | pyruvate kinase [EC:2.7.1.40] |
| FLJB16400 | rFLJB26655 | sc_FLJ_013271 | 100.0 | UDPglucose 6-dehydrogenase [EC:1.1.1.22] |

Note: FLJ, *Lonicera japonica* Thumb; rFLJ, *Lonicera japonica* Thunb. var. chinensis (Wats.)

**Supplement Table 7 PAL, CHS, HMGR and PK Gene Families in Arabidopsis and Grape**

| **Gene** | **Arabidopsis** | **Grape** | **Gene** | **Arabidopsis** | **Grape** |
| --- | --- | --- | --- | --- | --- |
| **PAL** | AT3G53260  AT2G37040  AT5G04230  AT3G10340 | GSVIVT0101514000117825944  GSVIVT0101512300117825931  GSVIVT0101512400117825932  GSVIVT0101513800117825942  GSVIVT0102429400117832537  GSVIVT0102429300117832536  GSVIVT0102429200117832535  GSVIVT0102430300117832543  GSVIVT0102430500117832544  GSVIVT0102429900117832540  GSVIVT0102430600117832545  GSVIVT0102429500117832538  GSVIVT0102431500117832547  GSVIVT0101625700117826722  GSVIVT0102570300117833642  GSVIVT0102521400117833253  GSVIVT0100614800117819761 | **CHS** | AT5G13930  AT4G34850  AT1G02050  AT4G00040 | GSVIVT0101056100117822688  GSVIVT0101057000117822693  GSVIVT0101058900117822704  GSVIVT0101058000117822705  GSVIVT0101055600117822686  GSVIVT0101056800117822692  GSVIVT0101055400117822684  GSVIVT0101057800117822696  GSVIVT0101057900117822697  GSVIVT0101055700117822687  GSVIVT0101057400117822695  GSVIVT0101058100117822699  GSVIVT0101058200117822700  GSVIVT0101058400117822702  GSVIVT0101057200117822694  GSVIVT0101058300117822701  GSVIVT0102621300117834017  GSVIVT0102622000117834018  GSVIVT0101058500117822703  GSVIVT0101058000117822698  GSVIVT0101056300117822689  GSVIVT0101056500117822690  GSVIVT0100052100117816993  GSVIVT0103296800117838809  GSVIVT0102410700117832386  GSVIVT0101821900117828239 |
| **Gene** | **Arabidopsis** | **Grape** | **Gene** | **Arabidopsis** | **Grape** |
| **HMGR** | AT1G76490  AT2G17370 | GSVIVT0102644400117884145  GSVIVT0102385200117832165  GSVIVT0101343500117824701 | **PK** | AT3G49160  AT3G52990  AT2G36580  AT3G55810  AT3G55650  AT3G25960  AT3G04050  AT5G08570  AT5G63680  AT5G56350  AT4G26390  AT3G22960  AT5G52920  AT1G32440 | GSVIVT0102870100117835737  GSVIVT0103374700117839405  GSVIVT0103468400117840078  GSVIVT0103359900117839281  GSVIVT0102984000117836509  GSVIVT0103337900117839105  GSVIVT0102550400117833484  GSVIVT0103704300117841785  GSVIVT0101807900117828138  GSVIVT0100314000117818308  GSVIVT0100287560117835782 |

**Supplement Table 8 Putative Enzyme Pool to Control the Active Compounds in Buds of *Lonicera japonica* Thumb**

| **Function** | **EC number** |
| --- | --- |
| 3-hydroxy-3-methylglutaryl-CoA reductase | 1.1.1.34 |
| 4-diphosphocytidyl-2-C-methyl-D-erythritol kinase | 2.7.1.148 |
| 4-hydroxy-3-methylbut-2-enyl diphosphate reductase | 1.17.1.2 |
| ATP citrate (pro-S)-lyase | 2.3.3.8 |
| beta-amylase | 3.2.1.2 |
| caffeoyl-CoA O-methyltransferase | 2.1.1.104 |
| chalcone synthase | 2.3.1.74 |
| dihydrolipoamide dehydrogenase | 1.8.1.4 |
| enolase | 4.2.1.11 |
| fatty acyl-ACP thioesterase B | 3.1.2.14/ 3.1.2.- |
| glucose-1-phosphate adenylyltransferase | 2.7.7.27 |
| isopentenyl-diphosphate delta-isomerase | 5.3.3.2 |
| malate dehydrogenase | 1.1.1.37 |
| omega-3 fatty acid desaturase (delta-15 desaturase) | 1.14.19.- |
| phenylalanine ammonia-lyase | 4.3.1.24 |
| phosphoglucomutase | 5.4.2.2 |
| pyruvate kinase | 2.7.1.40 |
| UDPglucose 6-dehydrogenase | 1.1.1.22 |
